# Measures of fragmentation of rest activity patterns: mathematical properties and interpretability based on accelerometer real life data

**DOI:** 10.1186/s12874-024-02255-w

**Published:** 2024-06-07

**Authors:** Ian Meneghel Danilevicz, Vincent Theodoor van Hees, Frank C. T. van der Heide, Louis Jacob, Benjamin Landré, Mohamed Amine Benadjaoud, Séverine Sabia

**Affiliations:** 1 Université Paris Cité, INSERM, U1153, CRESS, Epidemiology of Ageing and Neurodegenerative Diseases, 10 Av de Verdun, 75010 Paris, France; 2 Accelting, Almere, the Netherlands; 3 Institut de Radioprotection et de Sûreté Nucléaire (IRSN), 31 Av Division Leclerc, 92260 Fontenay-Aux-Roses, France; 4 Department of Epidemiology and Public Health, University College London, London, UK

**Keywords:** Circadian rhythm, Detrended fluctuation analysis, Inter-daily stability, Intradaily variability, Transition probability, Whitehall II cohort

## Abstract

Accelerometers, devices that measure body movements, have become valuable tools for studying the fragmentation of rest-activity patterns, a core circadian rhythm dimension, using metrics such as inter-daily stability (IS), intradaily variability (IV), transition probability (TP), and self-similarity parameter (named $\alpha$). However, their use remains mainly empirical. Therefore, we investigated the mathematical properties and interpretability of rest-activity fragmentation metrics by providing mathematical proofs for the ranges of IS and IV, proposing maximum likelihood and Bayesian estimators for TP, introducing the activity balance index (ABI) metric, a transformation of $\alpha$, and describing distributions of these metrics in real-life setting. Analysis of accelerometer data from 2,859 individuals (age=60-83 years, 21.1% women) from the Whitehall II cohort (UK) shows modest correlations between the metrics, except for ABI and $\alpha$. Sociodemographic (age, sex, education, employment status) and clinical (body mass index (BMI), and number of morbidities) factors were associated with these metrics, with differences observed according to metrics. For example, a difference of 5 units in BMI was associated with all metrics (differences ranging between -0.261 (95% CI -0.302, -0.220) to 0.228 (0.18, 0.268) for standardised TP rest to activity during the awake period and TP activity to rest during the awake period, respectively). These results reinforce the value of these rest-activity fragmentation metrics in epidemiological and clinical studies to examine their role for health. This paper expands on a set of methods that have previously demonstrated empirical value, improves the theoretical foundation for these methods, and evaluates their empirical use in a large dataset.

**Supplementary Information:**

The online version contains supplementary material available at 10.1186/s12874-024-02255-w.

## Introduction

A large number of human behaviours and physiological functions follow a circadian rhythmicity, such as for examples sleep/wake cycles, body temperature, and hormonal levels [1]. Circadian regulation of these processes is critical to maintaining homeostasis; prolonged disruptions are detrimental to health [2, 3], highlighting the importance of precise, scalable measures of human circadian rhythm (CR). Accelerometers, devices that record acceleration of the part of the body to which they are attached, have emerged as valuable tools to measure dimensions of CR based on movements in free-living conditions [4, 5].

An important dimension of CR is the fragmentation of rest-activity patterns over several consecutive days [6, 7]. Over time several metrics have been proposed to quantity the rest-activity fragmentation using accelerometry data. The first and now commonly used metrics are inter-daily stability (IS) and intradaily variability (IV). IS provides information on how constant the rest-activity pattern is between days and IV quantifies the fragmentation of activity pattern between consecutive hours over the observation period [6, 8]. Later the transition probability (TP) has been proposed to measure the likelihood of transitioning from a state of rest to a state of activity, or vice versa [7, 9]. Overall, the metrics described above are increasingly used in the context of fragmentation of rest-activity patterns but they can be used in different contexts involving series of dichotomous states, apart for IS and IV that are more specific to organisation of activity during a period of time. In parallel, the detrended fluctuation analysis (DFA) [10] initially used in genomics has been used to identify hidden patterns where activity fluctuations are used as proxy for rest-activity fragmentation [11, 12]. In DFA, the self-similarity parameter, also known as the scaling exponent or $\alpha$, is a key metric for description of time series, such as stationary and non-stationary time series, random noise and fractal noise, among others [13].

Although metrics of rest-activity fragmentation are increasingly used in the literature, mathematical properties of these metrics and their interpretation have not been entirely described. First, although the range of IS in [0, 1] and IV in [0, 2] has been suggested by van Someren et al. (1999) [14], no proper mathematical proof is available, limiting confidence in interpretability, particularly for extreme values. Second, Lim et al. (2011) [7] and Di et al. (2017) [9] have proposed different estimations of TP, both based on heuristic estimators, limiting their mathematical properties as compared to estimators based on maximum likelihood (ML) or Bayesian inference. In addition, the properties of these two estimators have not been compared. Third, interpretation of DFA-derived metrics is not straightforward. Finally, to our knowledge, only one study has shown the correlation between IS, IV, TP (based on Lim et al. (2011) definition [7]) and DFA within a unique sample, older adults living in residential facilities, limiting generalisability of findings [7].

In order to overcome limitations of the current evidence on rest-activity fragmentation metrics, the present study aims to 1) provide mathematical proof of the range of IS and IV, 2) propose a ML estimator, the gold standard of estimation, and a Bayesian estimator, with good properties, for TP, and 3) propose a new metric, that is a transformation of DFA-derived self-similarity parameter, named activity balance index (ABI), that reflects how balanced is the activity over several days, and 4) describe these metrics using data from the population-based Whitehall II accelerometer sub-study.

## Materials and methods

### Preliminary definitions

Rest-activity fragmentation metrics are calculated based on different time series derived from raw acceleration signals (Table 1). These time series differ as a function of units (eg minute or hour) and outcomes considered (acceleration, dichotomous state (rest/activity), or proportion of the epoch in a state). Here are some preliminary definitions of these time series.

**Table 1:** Key information of each time series

Time series	$\varvec{x} \varvec{=} \varvec{(x}_{\varvec{1}}\varvec{,\ldots ,}\varvec{x}_{\varvec{T}}\varvec{)}^{\varvec{\prime }}$	$\varvec{y} \varvec{=} \varvec{(y}_{\varvec{1}}\varvec{,\ldots , y}_{\varvec{T}}\varvec{)}^{\varvec{\prime }}$	$\varvec{z} \varvec{=} \varvec{(z}_{\varvec{1}}\varvec{,\ldots ,} \varvec{z}_{\varvec{P}}\varvec{)}^{\varvec{\prime }}$
Unit	1 min	1 min	1 hour
Total length	T=10080	T=10080	P=168
Outcome	acceleration	rest/activity	proportion
Threshold	none	40 m*g*	40 m*g*

#### Definition 1

For each individual, a discrete stochastic process representing the intensity of movement over a time period [0, *T*] is defined as $\{X_t\}_{t \in T}$, with $X_t \in [0, \delta _x]$, $\delta _x<\infty$, and *t* corresponds to an epoch. The observed time series is a vector represented as $\varvec{x} = (x_1, \dots , x_T)'$. In the case of accelerometry data, $x_t$ corresponds to the acceleration recorded at the $t^{th}$ epoch and $\delta _x$ is the maximum measurable record for $x_t$.

#### Definition 2

For each individual, a second stochastic process representing the active (*a*) and rest (*r*) states is defined as $\{Y_t\}_{t \in T}$, with $Y_t \in \{r,a\}$, where $Y_t = a$ if $X_t > \delta _y$, and $\delta _y$ is the threshold which separates active and rest based on the amount of acceleration per epoch. The observed time series is a vector represented as $\varvec{y} = (y_1, \dots , y_T)'$.

#### Definition 3

For each individual, a third stochastic process representing the proportion of active states per hour is defined as $\{Z_p\}_{p \in P}$, with $Z_p \in [0, 1]$, where $Z_p = \delta _z^{-1}\sum \nolimits _{t=1}^{\delta _z} I(y_{t + \delta _z(p-1)}=a)$, $I(\cdot )$ is an indicator function equal to one if the condition is true and zero otherwise, $\delta _z$ is the number of epochs which build 1 hour (eg if one epoch corresponds to 1 minute then $\delta _z = 60$), *p* corresponds to a period of 1 hour, and *P* is the total number of hours during the period [0, *T*]. The observed time series is a vector represented as $\varvec{z} = (z_1, \dots , z_P)'$.

### Data

The Whitehall II study is an ongoing prospective cohort study established in 1985-1988 among 10308 British civil servants with clinical examinations every four-five years since inception. A written informed consent for participation was obtained at each contact. Research ethics approval was obtained from the University College London ethics committee (latest reference number 85/0938). An accelerometer measure was added to the 2012-2013 wave of data collection (age range 60 to 83 years) for participants seen at the London clinic and those living in the south-eastern regions of England who underwent clinical examination at home. Participants were requested to wear a tri-axial accelerometer (GENEActiv Original; Activinsights Ltd, Kimbolton, UK) on their non-dominant arm for nine consecutive 24-hour days. Accelerometer data, sampled at 85.7Hz and expressed relative to gravity ($1g = 9.81m/s^2$), were processed using GGIR v2.9-0 [15]. The Euclidean norm minus one (ENMO) of raw acceleration was calculated and corrected for calibration error and non-wear time. These acceleration values were averaged over 60-second epochs and we used a 40 m*g* cut-point to differentiate between rest and active periods as previously done in studies using wrist-worn raw acceleration devices [16, 17]. This cut-point was proposed in a study to differentiate between inactive periods and activities of light or moderate-to-vigorous intensities where adult participants undertook series of activities in a laboratory and mimic postures and behaviours from free-living conditions [18]. This cut-point is in agreement with a more recent study among older adults that showed good classification accuracy based on oxygen consumption during nine laboratory-based activities of daily living [19].

Waking periods (ie, periods between waking and sleep onset) for each day were identified using an algorithm for sleep detection based on wrist movement along with self-reported sleep onset and waking time using a sleep diary. This algorithm has been previously described and evaluated against polysomnography data [20]. Data from waking onset on day 2 to same time on day 8 were retained, resulting in seven days of data. Non-wear time was detected using an algorithm that has been previously described [21], and for the present study 2859 participants who wore the accelerometer over the full seven consecutive days were included in analyses.

**Fig. 1:**
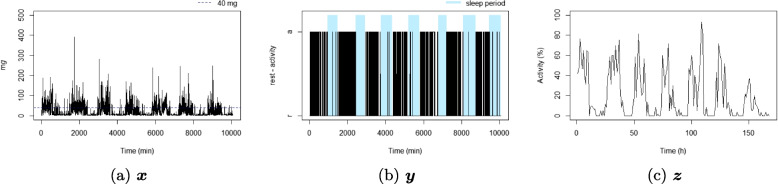
Example of three time series from the same individual

For each individual, three time series were considered: the time series corresponding to the 1-minute epoch acceleration over seven days $x_t$, $t = 1, \dots , 10080$ (the number of minutes over seven days), see Definition 1; the time series corresponding to the 1-minute epoch active state over seven days, $y_t$, $t = 1, \dots , 10080$, see Definition 2; and the time series corresponding to 1-hour proportion of active state over seven days, $z_p$, $p = 1, \dots , 168$ (the number of hours over seven days), see Definition 3. A summary of the three considered time series is available in Table 1, and an illustration is displayed in Fig. 1. For illustrative purposes, ten participant profiles were selected to highlight differences in metrics observed in real-life situations (six of them are displayed in Figs. 4, 5, 6, 7, 8, and 9 and four in the Supplementary material (figures S2-S5)). They were chosen based on their lowest or highest value in the metrics.

Measures of socio-demographic (age, sex, education) and health-related (body mass index (BMI), prevalent morbidities) factors were collected along with the accelerometer data in 2012-2013. Education was categorised as zero if the individual has less than secondary school education and one otherwise. BMI was based on measured weight and height (kg/m^2^), and the number of prevalent morbidities was assessed using clinical examinations in the study and linkage to electronic health records and includes coronary heart disease, stroke, heart failure.

A total of 4,880 individuals were invited to participate to the Whitehall accelerometer sub-study. Out of these, 4,282 agreed to wear the accelerometer and had no contraindications (allergy to plastic or travelling abroad). Among them, 2,859 individuals had complete data without any non-wear period for a continuous period of seven days corresponding to a total of 10,080 one-minute epochs. The mean age of the participants was 69.2 years, with a standard deviation (SD) of 5.7 years. A total of 602 were women (21.1%), 1170 (40.9%) had less than secondary school education level, 495 (17.3%) were currently employed, and 1140 (39.9%) had at least one morbidity. The mean body mass index (BMI) in the study sample was 26.7 (SD=4.3) kg/m^2^.

### Inter-daily stability and intradaily variability

#### Properties of IS and IV

IS measures how constant the rest-activity pattern is between days [8]. Considering that we measure *H* hours over *D* days, we have a total number of hours $P = H \times D$ over a full observation period. For IS, it is useful to organize the vector $\varvec{z}$ from Definition 3 in a matrix form as

$$\begin{aligned} \dot{\varvec{z}} = \left[ \begin{array}{ccc} z_{1,1} &{} \cdots &{} z_{1,H} \\ \vdots &{} &{} \vdots \\ z_{D,1} &{} \cdots &{} z_{D,H} \end{array}\right] , \end{aligned}$$

where $z_{d,h}$ is an element for the $d^{th}$ line and $h^{th}$ column, where $d = 1,\dots , D$ and $h = 1,\dots , H$. IS is computed as

$$\begin{aligned} \text {IS}(\varvec{z}) = \frac{P \sum \nolimits _{h=1}^{H} (\bar{z}_h-\bar{z})^2}{H \sum \nolimits _{p=1}^{P} (z_p-\bar{z})^2} = \frac{D \sum \nolimits _{h=1}^{H} (\bar{z}_h-\bar{z})^2}{ \sum \nolimits _{h=1}^{H}\sum \nolimits _{d=1}^{D} (z_{d,h}-\bar{z})^2}, \end{aligned}$$

where $\bar{z}_h = \frac{1}{D} \sum \nolimits _{d=1}^{D} z_{d,h}$ is the hour mean over the *D* days of measurement, and $\bar{z} = \frac{1}{P} \sum \nolimits _{p=1}^{P} z_p$ is the general mean over the full observed period.

IV represents the fragmentation of the rest-activity pattern over a long period, it measures the variability between consecutive hours (Fig. 1c) [8]. IV is calculated as

1 $$\begin{aligned} \text {IV}(\varvec{z}) = \frac{P \sum \nolimits _{p=2}^{P} (z_p-z_{p-1})^2}{(P-1) \sum \nolimits _{p=1}^{P} (z_p-\bar{z})^2}. \end{aligned}$$

Some mild conditions should be established to derive the properties of IS and IV metrics. They are:

1. $Z_p$ follows an autoregressive model of order 1 (AR(1) model) as
  $$\begin{aligned} Z_p = \mu + \phi Z_{p-1} + \epsilon _p, \end{aligned}$$
  where $\mu$ is the mean of the stochastic process, $\phi$ is a fixed but unknown parameter with $|\phi |<1$, $\epsilon _p$ is a Gaussian noise.
2. $$0 \le \phi < 1$$ and $$P \rightarrow \infty$$.

The assumption (A1) is required to define the IV range, because we need to determine the relationship between $Z_p$ and $Z_{p-1}$, and the AR(1) model is a very simple and flexible model, which can fit several different real situations. Although, we assume a stationary process, see unit root conditions in Dickey and Fuller (1979) [22]. The assumption (A2) is imposed to guarantee a positive auto-correlation $0 \le \phi$, and a long period of observation, *P*, of the time series.

##### Theorem 1

Given a stochastic process $\{Z_p\}_{p \in P}$, $\text {IS}(\varvec{z}) \in [0,1]$.

##### Theorem 2

Given a stochastic process $\{Z_p\}_{p \in P}$ and under assumption (A1), $\text {IV}(\varvec{z}) \in [0,\infty )$.

##### Theorem 3

Given a stochastic process $\{Z_p\}_{p \in P}$ and under assumptions (A1) and (A2), $\lim _{P \rightarrow \infty }(\text {IV}(\varvec{z})) \in [0,2]$.

The proofs of these theorems are provided in the Supplementary material (Section 1).

#### Interpretation of IS and IV

In the demonstration of Theorem 1 (Supplementary material - Section 1), we showed that a higher value of IS reflects a rest-active pattern that is more constant between days, see (Table 2).

**Table 2:** Range, interpretability, strengths and limitations of rest-activity fragmentation metrics

Inter-daily stability (IS)	
• Measure: how constant is rest-activity pattern between days.	
• Range: [0, 1]	
• Interpretation: higher values represent more constant rest-activity pattern.	
• Strengths: it uses smooth data (over hours of the day) and is less sensitive to extreme values.	
• Limitations: it depends on differentiation between rest/activity states for which no standards exist.	
Intradaily variability (IV)	
• Measure: the variability in activity hour by hour throughout the days.	
• Range: $[0, \infty )$	
• Interpretation: higher values represent more fragmented rhythm, values higher than two means ultradian rhythm or small sample size.	
• Strengths: it uses smooth data (over hours of the day) making it less sensitive to extreme values.	
• Limitations: it depends on differentiation between rest/activity states for which no standards exist.	
Transition probability (TP)	
• Measure: the TP from rest to activity (or activity to rest).	
• Range: (0, 1]	
• Interpretation:	
– $\text {TP}_{ra,\texttt{w}}$ : higher values correspond to higher TP from rest to activity when in a rest period during the day, denoting a more fragmented rest/sedentary behaviour during the day.	
– $\text {TP}_{ra,\texttt{s}}$ : higher values correspond to higher TP from rest to activity when in an rest period during the night, denoting a more fragmented rest during the night.	
– $\text {TP}_{ar,\texttt{w}}$ : higher values correspond to higher TP from activity to rest when in an active period during the day, denoting a more fragmented activity pattern during the day.	
– $\text {TP}_{ar,\texttt{s}}$ : higher values correspond to higher TP from activity to rest when in an active period during the night, denoting a higher propensity to go back to rest when active during the night.	
• Strengths: it is based on dichotomous state (rest or activity) at the bout level, making it less sensitive to extreme values. It is defined separately during awake (day) and sleep (night) periods, allowing inference on the relevance of fragmentation of rest and activity separately for both periods.	
• Limitations: it depends on a cut-point to differentiate rest from activity states.	
Self-similarity parameter (${\alpha }$)	
• Measure: the self-similarity of acceleration signal over the observation period.	
• Range: (0, 2)	
• Interpretation: values in the range (0, 1) means stationary motion behaviour. Values in the range (1, 2) means nonstationary motion behaviour. There are critical points as: 0.5 means random noise, 1 fractal noise and 1.5 random walk.	
• Strengths: it considers the full activity distribution and is not dependent on the choice of a cut-point to differentiate rest from activity states.	
• Limitations: it is sensitive to extreme values that could be observed in the data. The interpretation requires mathematical knowledge.	
Activity balance index (ABI)	
• Measure: how the activity is balanced over the observation period.	
• Range: (0, 1]	
• Interpretation: higher values represent more balanced movement behaviour.	
• Strengths: it considers the full activity distribution and is not dependent on the choice of a cut-point to differentiate rest from activity states. It is easy to interpret.	
• Limitations: it is sensitive to extreme values that could be observed in the data.	

In the demonstration of Theorem 2 (Supplementary material - Section 1), we showed that 1) if $\phi$ goes to one (perfect autocorrelation), then IV goes to zero, reflecting a low rest-activity fragmentation between hours; 2) if $\phi$ goes to zero (uncorrelated random noise), then IV goes to two, representing a high rest-activity fragmentation between hours; 3) in some specific cases, IV can be greater than two, this can occur when the sample size *P* is too small, or $\phi < 0$ which may be seen in ultradian rhythm, which means that the rhythm cycle lasts less than a day [14], or in the case of use of high frequency data [23]. These statements agree with the previous claims given by van Someren et al. (1999) about IS and IV [14]. Some authors use IV based on the $\varvec{x}$ time series [24–26]. In that case, the present properties do not hold anymore as the assumptions (A1) and (A2) are verified exclusively for $\varvec{z}$.

### Transition probability

#### Properties of TP

The TP in dichotomous stochastic processes represents the probability of a state change given a period of time spent in a specific state. A formal characterization of TP of changes in rest/activity state is given in Definition 4.

##### Definition 4

For each individual, given a stochastic process determined by Definition 2 the TP from *r* to *a* given an uninterrupted period of rest with length equal to *s* is

$$\begin{aligned} \pi _{ra}(s) = \mathcal {P} (Y_t=a|Y_{t-1}=r,\dots , Y_{t-s} = r), \end{aligned}$$

and the TP from *a* to *r* given an uninterrupted period of activity with length equal to *s* is

$$\begin{aligned} \pi _{ar}(s) = \mathcal {P} (Y_t=r|Y_{t-1}=a,\dots , Y_{t-s}=a). \end{aligned}$$

The two conditional probabilities from Definition 4 are proposed by Lim et al. (2011) [7]. The specific cases of $s=1$ returns two probabilities proposed by Di et al. (2017) [9]. We aim to propose a ML estimator to TP because if the model assumptions are aligned, there is no better estimation than ML, being a gold standard. Although, if there is available knowledge, we can aggregate this information and build a Bayesian estimator that is even more accurate than ML. Beforehand, some notations need to be introduced for readability.

##### Definition 5

$\varvec{r} = (r_1, \dots , r_{n_{r}})'$ is a $n_{r}$-vector that records the length of each consecutive bout of rest, where $n_r$ is the number of bouts of rest $(n_r \le T)$ so that $r_1$ is the length of the first bout of rest, $r_2$ of the 2^nd^ bout of the rest, and $r_{n_r}$ the length of the last bout of rest. $T_{r} = \sum \nolimits _{i=1}^{n_{r}}r_i$ is the total length of rest (in epochs unit), $r_i \in \{1, \dots , S_r\}$, $S_r$ is the duration of the longest bout of rest.

##### Definition 6

$\varvec{a} = (a_1, \dots , a_{n_{a}})'$ is a $n_{a}$-vector that records the length of each consecutive bout of activity, where $n_a$ is the number of bouts of activity $(n_a \le T)$. $T_{a} = \sum \nolimits _{i=1}^{n_{a}}a_i = T -T_{r}$ is the total length of activity (in epochs unit), $a_i \in \{1, \dots , S_a\}$, $S_a$ is the duration of the longest bout of activity.

Here are two assumptions:

1. The stochastic process $\{Y_t\}_{t \in T}$ is stationary.
2. The stochastic process $\{Y_t\}_{t \in T}$ has a finite memory equal to $s \ge 1$.

##### Theorem 4

Given a stochastic process $\{Y_t\}_{t \in T}$, under assumptions (B1) and (B2), the ML estimators of $\pi _{ra}(s)$ and $\pi _{ar}(s)$ are $\hat{\pi }_{ra}(s)_{ML} = \frac{\sum \nolimits _{i=1}^{n_{r}} I(r_i \ge s)-I(y_T=r)}{\sum \nolimits _{i=1}^{n_{r}}(r_i-s+1) I(r_i \ge s)-I(y_T=r)}$ and $\hat{\pi }_{ar}(s)_{ML} = \frac{\sum \nolimits _{i=1}^{n_{a}} I(a_i \ge s)-I(y_T=a)}{\sum \nolimits _{i=1}^{n_{a}}(a_i-s+1) I(a_i \ge s)-I(y_T=a)}$, for $s=1,\dots ,S_r - 1$, and $s=1,\dots ,S_a - 1$, respectively.

##### Corollary 1

Given a stochastic process $\{Y_t\}_{t \in T}$, under assumptions (B1) and (B2), the ML estimators of $\pi _{ra}(1)$ and $\pi _{ar}(1)$ are $\hat{\pi }_{ra}(1)_{ML} = \frac{n_{r}-I(y_T=r)}{T_{r}-I(y_T=r)}$ and $\hat{\pi }_{ar}(1)_{ML} = \frac{n_{a}-I(y_T=a)}{T_{a}-I(y_T=a)}$.

##### Corollary 2

Given a stochastic process $\{Y_t\}_{t \in T}$, under assumptions (B1) and (B2), the Bayesian estimators of $\pi _{ra}(1)$ and $\pi _{ar}(1)$ are $\hat{\pi }_{ra}(1)_{B} = \frac{n_{r}-I(y_T=r)+\lambda }{T_{r}-I(y_T=r)+\lambda }$ and $\hat{\pi }_{ar}(1)_{B} = \frac{n_{a}-I(y_T=a)+\lambda }{T_{a}-I(y_T=a)+\lambda }$, for any hyperparameter $\lambda >0$.

The proof of Theorem 4 is provided in Supplementary material - Section 1 using as a main argument the properties of a Bernoulli stochastic process [27]. Also the proof of Corollary 1 is given in Supplementary material - Section 1. The proof of the Corollary 2 is a direct application from Corollary 1 for a Binomial model. Then the Beta-Binomial posterior estimator is a well-known result ([28], page 104).

Corollary 1 makes evident the intuitive relation between the number of transitions per total time in a specific state, which is a gain in terms of interpretability. In the Bayesian estimator introduced in Corollary 2, $\lambda$, present both in the numerator and denominator, is a pre-specified hyperparameter to the Beta prior distribution for the transition probability. This parameter allows the Bayesian estimator to always exist even if any of $T_{r}$ or $T_{a}$ is zero. If $\lambda =1$, corresponding to the Uniform distribution prior, we assume that in a sequence of nights there is at least one epoch of activity and in a sequence of days there is at least one epoch of rest. Other values might be explored such as $\lambda =0.5$ corresponding to the Horseshoe prior [29], or $\lambda =10^{-6}$ which returns a numerically insignificant difference between ML and Bayesian estimators. Larger values than one for $\lambda$ may not be relevant in this context as they are likely to deviate too much from the ML estimators.

##### Remark 1

Even without any assumption about the stochastic process in terms of memory and stationary, some nonparametric measures are avaliable as the reciprocal average duration (RAD) of rest, $\text {RAD}_r$, and the RAD of activity, $\text {RAD}_a$, which are defined as

2 $$\begin{aligned} \text {RAD}_{r} = \frac{n_{r}}{T_{r}}, \; \text {RAD}_{a} = \frac{n_{a}}{T_{a}}. \end{aligned}$$

This metric appears in previous work [30], but it was used to approximate the target probabilities $\pi _{ra}(1)$ and $\pi _{ar}(1)$ [9]. If $y_T = r$, then $\text {RAD}_{a} = \hat{\pi }_{ar}(1)_{ML}$ and $\text {RAD}_{r} > \hat{\pi }_{ra}(1)_{ML}$ as $T_{r} > n_{r}$; if $y_T = a$, then $\text {RAD}_{r} = \hat{\pi }_{ra}(1)_{ML}$ and $\text {RAD}_{a} > \hat{\pi }_{ar}(1)_{ML}$ as $T_{a} > n_{a}$.

Let us give a hypothetical example for a small sample size $T=15$ as $\varvec{y} = (a,a,a,r,r,a,r,a,a,a,r,r,a, r,r)'$ to illustrate the difference between $\hat{\pi }_{ra}(1)_{ML}$, $\hat{\pi }_{ra}(1)_{B}$ and $\text {RAD}_{r}$, as well as $\hat{\pi }_{ar}(1)_{ML}$, $\hat{\pi }_{ar}(1)_{B}$ and $\text {RAD}_{a}$, with hyperparameter $\lambda =0.5$. Here we have $\varvec{r} = (2,1,2,2)'$ and $\varvec{a} = (3,1,3,1)'$, this corresponds to $n_r=4$, $n_r-I(y_T=r)=3$, $n_a=4$, $n_a-I(y_T=a)=4$, $T_r=7$, $T_r-I(y_T=r)=6$, $T_a=8$, $T_a-I(y_T=a)=8$. So we have three changes ($n_r-I(y_T=r)$) in six opportunities ($T_r-I(y_T=r)$) ie 50% of transitions from *r* to *a* by the ML estimator, the $\text {RAD}_{r}$ inflates this result to 57% by adding a transition for the last observation, but actually we don’t know what would happen in $y_{16}$. From *a* to *r*, $\text {RAD}_a$ and ML estimators are the same, and Bayesian estimator is also really close. For convenience, these values are available in Table 3.

**Table 3:** Hypothetical example

Method	$\text {RAD}_{r}$	$\hat{\pi }_{ra}(1)_{ML}$	$\hat{\pi }_{ra}(1)_{B}$	$\text {RAD}_{a}$	$\hat{\pi }_{ar}(1)_{ML}$	$\hat{\pi }_{ar}(1)_{B}$
Numerator	4	3	3.5	4	4	4.5
Denominator	7	6	6.5	8	8	8.5
Estimation	0.57	0.50	0.54	0.50	0.50	0.53

The conditional probabilities $\pi _{ar}(1)$ and $\pi _{ra}(1)$ are more convenient to interpret than $\pi _{ar}(s)$ and $\pi _{ra}(s)$. In the aim of summarizing TP, Lim et al. (2011) proposed a bounded average calculated by LOWESS smoothing over a range of *s* values [7]. This method requires to determine the boundary of the *s* values for which there is not a straightforward method. In the application part of this paper, we chose $s=1$ and partitioned the observed vector $\varvec{y}$ in waking and sleep periods as described in Remark 2.

##### Remark 2

We propose a Bayesian estimator that compared to ML or RAD, avoids to have values that cannot be computed in case of no time spent in a state (denominator null). For an epidemiological motivation, we split these metrics by wake and sleep windows (that is the period between waking and sleep onset (wake), and between sleep and next waking for the day to start (sleep), respectively), as

$$\begin{aligned} \text {TP}_{ra, \texttt{w}} = \frac{n_{r, \texttt{w}}-I(y_T=r)+\lambda }{T_{r, \texttt{w}}-I(y_T=r)+\lambda }, \; \;\text {TP}_{ra, \texttt{s}} = \frac{n_{r, \texttt{s}}-I(y_T=r)+\lambda }{T_{r, \texttt{s}}-I(y_T=r)+\lambda }, \end{aligned}$$

the TP from rest to active period during the waking window, and the TP from rest to active period during the sleep window, respectively, and

$$\begin{aligned} \text {TP}_{ar, \texttt{w}} = \frac{n_{a, \texttt{w}}-I(y_T=a)+\lambda }{T_{a, \texttt{w}}-I(y_T=a)+\lambda }, \; \; \text {TP}_{ar, \texttt{s}} = \frac{n_{a, \texttt{s}}-I(y_T=a)+\lambda }{T_{a, \texttt{s}}-I(y_T=a)+\lambda }, \end{aligned}$$

the TP from active to rest period during the waking window, and the TP from active to rest period during the sleep window, respectively, where $\lambda \in (0,1]$, $n_{a, \texttt{w}}$ is the number of bouts of activity during the awake time, $n_{a, \texttt{s}}$ is the number of bouts of activity during the sleep time, $n_{r, \texttt{w}}$ is the number of bouts of rest during the awake time, $n_{r, \texttt{s}}$ is the number of bouts of rest during the sleep time, $T_{r, \texttt{w}}$ is the total rest time during the awake time, $T_{r, \texttt{s}}$ is the total rest time during the sleep time, $T_{a, \texttt{w}}$ is the total activity time during the awake time, and $T_{a, \texttt{s}}$ is the total activity time during the sleep time.

#### Interpretation of TP

When using a small $\lambda$ and a long period of observation, higher $\text {TP}_{ra, \texttt{w}}$ corresponds to more transitions from rest to active periods during the awake window, reflecting a more fragmented pattern of rest, higher $\text {TP}_{ar, \texttt{w}}$ corresponds to more transitions from active to rest periods during the awake window, denoting a more fragmented pattern of activity. A similar interpretation applies to the metrics defined during the sleep window (Table 2). In case of one state not being observed during a window as for example no activity at all during the sleep window, the TP exists and transition from this unobserved state to the observed state is equal to 1. This means that in case this person moves to this unobserved state, it is highly likely that he or she will return to the observed state quickly.

### Detrended fluctuation analysis

#### Introduction to DFA

The DFA is a powerful analytical tool for time series analysis initially proposed by Peng et al. (1994) to analyse long-term correlation of nucleotides [10]. More recently it has been used in the context of movement behaviour to quantity fractal fluctuations in activity over a range of time scales [12, 31]. In practise, it aims to evaluate to which extent the activity pattern (in terms of temporal and structural properties) is similar at different time scales. Estimating the self-similarity parameter allows differentiating stationary and nonstationary stochastic processes and identifying white, pink (fractal), or brown noise patterns. These key properties might be hidden in complex time series, but DFA is a way to reveal them.

Let us consider a bounded stochastic process $\{X_t\}_{t \in T}$ from Definition 1. Take the accumulated signal with zero mean as

$$\begin{aligned} c_t = \sum \limits _{i=1}^{t}(x_i - \bar{x}), \; t \le T, \end{aligned}$$

where $\bar{x} = T^{-1}\sum \nolimits _{t=1}^{T}x_t$. Divide $\varvec{c} = (c_1, \dots , c_T)'$ in *B* nonoverlapping boxes of equal *n*-size as $\varvec{c}_1 = (c_1, \dots , c_n)'$, $\varvec{c}_2 = (c_{n+1}, \dots , c_{2n})'$, until $\varvec{c}_B = (c_{(B-1)n+1}, \dots , c_{Bn})'$. For each box, we fit a polynomial of order *l*, eg, the polynomial for the $j^{th}$ box is fit using an ordinary least squares regression as

3 $$\begin{aligned} f_t(n) = \hat{\beta }_0 + \hat{\beta }_1 t + \dots + \hat{\beta }_l t^l, \end{aligned}$$

where $t= (j-1)n+1, \dots , jn$. In the application section of this paper, we restricted our analysis to $l=1$ as in previous works [26, 32]. In the Supplementary material (Section 3), we replicated the analysis using $l=2$ for comparison. A polynomial order higher than two is not expected to change the results [33]. Note that $\varvec{\beta }=(\beta _0, \dots , \beta _l)'$ is different to each $j^{th}$ box and each *n*-size, consequently $f_t(n)$ depends of *t* and *n*. To detrend the integrated time series, ie, remove the trend of $c_t$, we take the difference of each pair $c_t$ and $f_t(n)$. For a given *n*-size box, the root mean square fluctuation is

4 $$\begin{aligned} F(n) = \sqrt{\frac{1}{T} \sum \limits _{t=1}^{T}(c_t - f_t(n))^2}. \end{aligned}$$

**Fig. 2:**
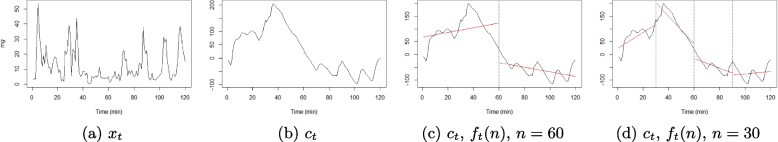
Example of DFA procedure

Repeat the operation for a broad range of *n*-size boxes, eg, Mesquita et al. (2020) recommend taking a sample on the grid between $4 \le n \le T/4$ [34, 35]. The Fig. 2 displays the steps of DFA for two *n*-size boxes, the first with 60 minutes (Fig. 2c) and the second with 30 minutes (Fig. 2d).

#### Summary statistic for DFA and interpretation

Instead of displaying a function of *F*(*n*) for a grid of *n*, we can summarize this information by the self-similarity parameter. The root mean square fluctuation in (4) is proportional to the *n*-size, $F(n) \propto n^\alpha$, where $\alpha$ is called the scaling exponent or self-similarity parameter, which is estimated using

$$\begin{aligned} \log (F(n)) = \mu + \alpha \log (n) + \epsilon _n, \; 4 \le n \le T/4, \end{aligned}$$

where $\epsilon _n$ follows an independent Gaussian error, $\mu$ is an intercept, and an ordinary least squares regression (OLS) is used to calculate $\hat{\alpha }$.

The interpretation of $\alpha$ is quite precise, but requires much mathematical jargon. Given a stochastic process as determined by Definition 1, the self-similarity parameter belongs to the range $0< \alpha <1$ for stationary stochastic processes, and $1< \alpha <2$ for nonstationary as proofed by Løvsletten (2017) [36]. Some critical values of the scaling exponent are of distinct mathematical importance as $\alpha =0.5$ means that the stochastic processes is white noise, $\alpha =1$ is related to pink or fractal noise, $\alpha =1.5$ is the case of a random walk [32].

#### Activity balance index: a new DFA-derived metric

Given previous empirical results, Hausdorff et al. (1996) hypothesized that many biological systems present a fractal nature, ie, $\alpha =1$ [37]. A further hypothesis that healthy people presents fractal noise for heart and walking rates has been elaborated by Peng et al. (2000) [38]. In the context of activity behaviour, we have introduced a novel metric named activity balance index (ABI), that measures how the activity over the observed period is balanced, higher values reflect a more balanced pattern of activity. It is a transformation of $\hat{\alpha }$ as

5 $$\begin{aligned} \text {ABI}(\hat{\alpha }) = \exp \left\{ \frac{-|\hat{\alpha }-1|}{\exp (-2)} \right\} , \end{aligned}$$

where $\hat{\alpha } \in (0,2)$. If $\hat{\alpha }$ goes to one, then $|\hat{\alpha }-1|$ goes to zero and $\text {ABI}(\hat{\alpha })$ goes to one. On the other direction, as $\hat{\alpha }$ goes to two or zero, which are the extremes for $\alpha$ [36], $|\hat{\alpha }-1|$ goes to one and $\text {ABI}(\hat{\alpha })$ goes to 0.0006. The ABI has two advantages: it penalizes the scattering of $\hat{\alpha }$ in both directions and spreads its values over a large range between (0.0006, 1] or (0, 1] for simplicity.

We introduced the ABI that focusses on the fractal noise nature of the signal to evaluate how the activity is balanced over the observation period. If fractal noise represents an optimum balance for activity behaviour, then healthy individuals would present higher values for their ABI metric than unhealthy people (Table 2). Both $\hat{\alpha }$ and ABI are influenced by the choice of the epoch lengths, larger epoch values will naturally tend to smooth acceleration signal, implying lower chance of observing a fractal noise (that is $\hat{\alpha }$ and ABI closer to one).

### Strengths and limitations of IS, IV, TP and DFA

The strengths and limitations of rest-activity fragmentation metrics are summarized in Table 2.

#### Remark 3

The metrics mentioned above rely on transitions between rest and active states and do not capture other dimensions of the circadian rhythm. These metrics can be used in complement to other circadian rhythm variables that capture different aspects of the rhythm such as timing or amplitude.

## Results

**Fig. 3:**
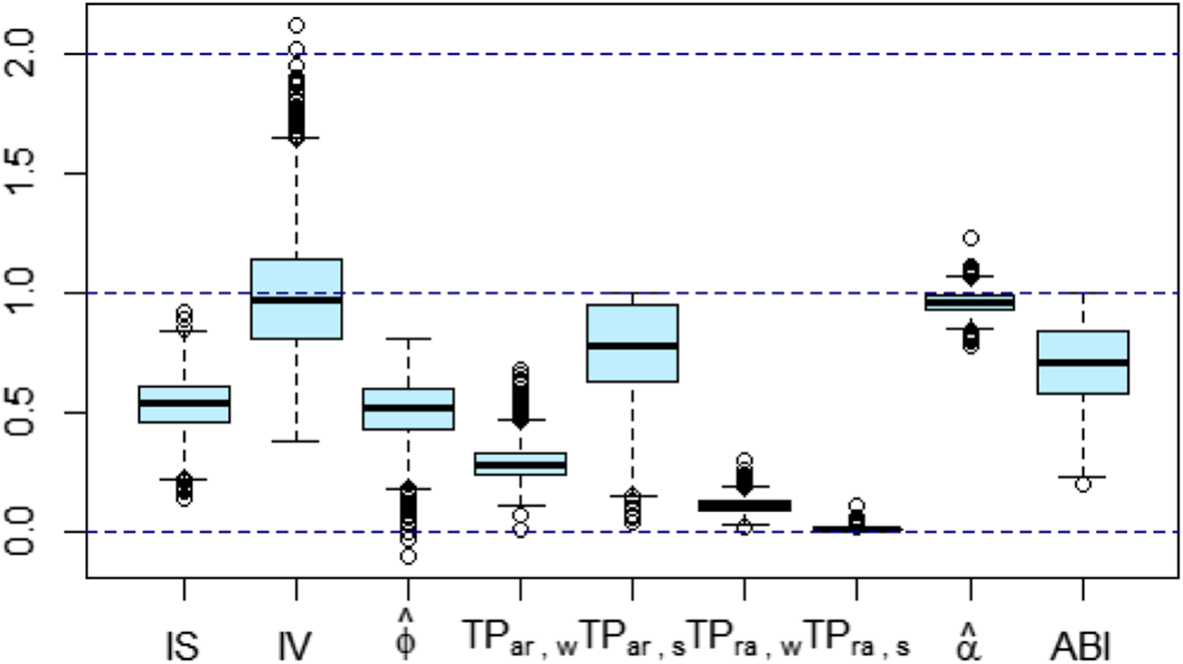
Boxplot of inter-daily stability (IS), intradaily variability (IV), estimated autocorrelation parameter of AR(1) model ($\hat{\phi }$), transition probability (TP) from activity to rest during the awake period ($\text {TP}_{ar,\texttt{w}}$), TP from activity to rest during the sleep period ($\text {TP}_{ar,\texttt{s}}$), TP from rest to activity during the awake period ($\text {TP}_{ra,\texttt{w}}$), TP from rest to activity during the sleep period ($\text {TP}_{ra,\texttt{s}}$), estimated self-similarity ($\hat{\alpha }$), and activity balance index (ABI)

Figure 3 shows the distribution of IS, IV, TP, $\hat{\alpha }$ and ABI in the total sample. All empirical ranges are within the theoretical ones proposed in Table 2. For IV, two individuals have a value that exceeds two, these outliers correspond to two of the three individuals whose $\hat{\phi }$ value is not within the [0, 1] interval, suggesting a minority of cases with ultradian rhythm in the dataset.

When examining how rest-activity fragmentation metrics differ by sex (Table 4), we found that men have on average a less constant rest-activity pattern as denoted by smaller IS compared to women (0.529 vs 0.546, $p=0.001$). During the day, men tend to transition less from rest to active periods while during the night men are more likely to transition from active to rest periods as indicated by lower $\text {TP}_{ra,\texttt{w}}$ ($p<0.001$) and higher $\text {TP}_{ar,\texttt{s}}$ ($p<0.001$) than in women. Finally, on average they tend to have a less balanced activity behaviour than women as shown by lower $\hat{\alpha }$ ($p=0.022$) and ABI ($p<0.001$).

Fewer differences were observed as a function of age, although we found that older people tend to have a more fragmented rest-activity pattern (IV 1.039 vs 0.946 for age $\ge$ 70 vs <70), to transition more from activity to rest during waking periods ($\text {TP}_{ar,\texttt{w}}$ 0.305 vs 0.273), and to transition less from rest to active periods during the day ($\text {TP}_{ra,\texttt{w}}$ 0.098 vs 0.109), but more during the night ($\text {TP}_{ra,\texttt{s}}$ 0.08 vs 0.007); all $p<0.001$.

**Table 4:** Mean (SD) of inter-daily stability (IS), intradaily variability (IV), transition probability (TP) from activity to rest during the awake period ($\text {TP}_{ar,\texttt{w}}$), TP from activity to rest during the sleep ($\text {TP}_{ar,\texttt{s}}$), TP from rest to activity during the awake ($\text {TP}_{ra,\texttt{w}}$), TP from rest to activity during the sleep ($\text {TP}_{ra,\texttt{s}}$), estimated self-similarity parameter ($\hat{\alpha }$), and activity balance index (ABI) in the total population (*N*=2857), by sex and age groups

	all	men	women		age $<70$	age $\ge 70$	
N	2859	2257	602		1717	1142	
	mean (SD)	mean (SD)	mean (SD)	*p*-value	mean (SD)	mean (SD)	*p*-value
IS	0.533 (0.116)	0.529 (0.116)	0.546 (0.116)	0.001	0.534 (0.114)	0.531 (0.119)	0.442
IV	0.983 (0.251)	0.987 (0.254)	0.970 (0.241)	0.120	0.946 (0.237)	1.039 (0.261)	$<.001$
$\text {TP}_{ar,\texttt{w}}$	0.285 (0.076)	0.285 (0.075)	0.286 (0.080)	0.931	0.273 (0.067)	0.305 (0.084)	$<.001$
$\text {TP}_{ar,\texttt{s}}$	0.765 (0.194)	0.780 (0.193)	0.711 (0.189)	$<.001$	0.762 (0.194)	0.770 (0.195)	0.232
$\text {TP}_{ra,\texttt{w}}$	0.105 (0.033)	0.104 (0.032)	0.110 (0.034)	$<.001$	0.109 (0.031)	0.098 (0.033)	$<.001$
$\text {TP}_{ra,\texttt{s}}$	0.007 (0.005)	0.007 (0.005)	0.007 (0.004)	0.896	0.007 (0.004)	0.008 (0.006)	$<.001$
$\hat{\alpha }$	0.956 (0.046)	0.955 (0.048)	0.959 (0.037)	0.022	0.957 (0.047)	0.954 (0.043)	0.220
ABI	0.700 (0.169)	0.693 (0.172)	0.730 (0.152)	$<.001$	0.699 (0.171)	0.703 (0.166)	0.573

All *p*-values come from an ANOVA test

Table 5 shows one fitted multivariate regression for each standardized rest-activity fragmentation metric. Being a woman, aged around 70 years old (see Figure S1 in Supplementary additional results for association with age), with lower educational level, not currently employed, having lower BMI and less prevalence morbidities were associated with a more constant rest-activity pattern (all $p<0.05$). The same variables (except for sex) were associated with IV, but in the opposite direction, denoting a less fragmented rest-activity pattern. $\text {TP}_{ar,\texttt{w}}$ was associated with all socio-demographic and health-related factors (except for sex and employment status), and $\text {TP}_{ar,\texttt{s}}$, in a complementary way, was only significantly associated with sex and employment status. Higher $\text {TP}_{ra,\texttt{w}}$ was associated with being a woman, lower BMI and less morbidities while higher $\text {TP}_{ra,\texttt{s}}$ was associated with higher BMI and more prevalent morbidities. Both $\hat{\alpha }$ and ABI were associated with all socio-demographic (except education) and health-related factors.

**Table 5:** Association of socio-demographic and health-related factors with standardized rest-activity fragmentation metrics, results from multivariate linear regressions

	Coeff.	(95% CI)	Coeff.	(95% CI)	Coeff.	(95% CI)	Coeff.	(95% CI)
	IS		IV		$\hat{\alpha }$		ABI	
Age *per 10 years*	2.535	(0.896, 4.175)^a^	-4.861	(-6.464, -3.258)^a^	3.721	(2.073, 5.369)^a^	2.890	(1.235, 4.546)^a^
Age^2^ *per 10 years*	-0.182	(-0.298, -0.066)^a^	0.373	(0.259, 0.487)^a^	-0.267	(-0.384, -0.150)^a^	-0.204	(-0.321, -0.086)^a^
Women	0.154	(0.064, 0.243)^a^	-0.063	(-0.150, 0.024)	0.110	(0.020, 0.200)^a^	0.219	(0.129, 0.309)^a^
Currently employed	-0.327	(-0.424, -0.229)^a^	0.136	(0.041, 0.232)^a^	-0.178	(-0.276, -0.080)^a^	-0.101	(-0.200, -0.003)^a^
High education	-0.112	(-0.188, -0.037)^a^	0.184	(0.110, 0.257)^a^	-0.064	(-0.140, 0.011)	-0.076	(-0.151, 0.000)
BMI *per 5 kg/m* ^2^	-0.168	(-0.211, -0.126)^a^	0.180	(0.139, 0.221)^a^	-0.152	(-0.194, -0.110)^a^	-0.127	(-0.169, -0.084)^a^
Number of morbidities	-0.085	(-0.135, -0.036)^a^	0.066	(0.018, 0.115)^a^	-0.073	(-0.123, -0.024)^a^	-0.056	(-0.106, -0.006)^a^
	$\text {TP}_{ar,\texttt{w}}$		$\text {TP}_{ar,\texttt{s}}$		$\text {TP}_{ra,\texttt{w}}$		$\text {TP}_{ra,\texttt{s}}$	
Age *per 10 years*	-2.874	(-4.452, -1.295)^a^	-0.777	(-2.439, 0.884)	1.026	(-0.573, 2.624)	-0.828	(-2.479, 0.824)
Age^2^ *per 10 years*	0.234	(0.122, 0.346)^a^	0.059	(-0.059, 0.177)	-0.094	(-0.208, 0.019)	0.075	(-0.042, 0.192)
Women	-0.024	(-0.110, 0.062)	-0.346	(-0.437, -0.256)^a^	0.241	(0.154, 0.328)^a^	-0.016	(-0.106, 0.074)
Currently employed	-0.011	(-0.105, 0.083)	-0.106	(-0.204, -0.007)^a^	0.036	(-0.059, 0.131)	0.064	(-0.034, 0.162)
High education	0.088	(0.015, 0.160)^a^	0.023	(-0.053, 0.099)	-0.066	(-0.140, 0.007)	-0.053	(-0.128, 0.023)
BMI *per 5 kg/m* ^2^	0.228	(0.187, 0.268)^a^	-0.041	(-0.083, 0.002)	-0.261	(-0.302, -0.220)^a^	0.050	(0.007, 0.092)^a^
Number of morbidities	0.113	(0.066, 0.161)^a^	-0.030	(-0.080, 0.021)	-0.086	(-0.134, -0.038)^a^	0.114	(0.064, 0.164)^a^

^a^means significant at 0.95 confidence level, estimated coefficient (Coeff.), 95% confidence interval (95% CI), high education (secondary school or above), morbidities (number of prevalent morbidities among: coronary heart disease, stroke, heart failure, cancer, arthritis, chronic obstructive pulmonary disease, depression, Parkinson’s disease and dementia)

Table 6 presents Pearson’s correlation coefficients between IS, IV, TPs, $\hat{\alpha }$, and ABI metrics. We observe one moderate correlation between $\hat{\alpha }$ and ABI that is expected as ABI is a transformation of $\hat{\alpha }$. All the remaining correlations are considered fair or poor [39].

**Table 6:** Pearson’s correlation between inter-daily stability (IS), intradaily variability (IV), transition probability (TP) from activity to rest during the awake period ($\text {TP}_{ar,\texttt{w}}$), TP from activity to rest during the sleep ($\text {TP}_{ar,\texttt{s}}$), TP from rest to activity during the awake ($\text {TP}_{ra,\texttt{w}}$), TP from rest to activity during the sleep ($\text {TP}_{ra,\texttt{s}}$), estimated self-similarity parameter ($\hat{\alpha }$), and activity balance index (ABI)

	IS	IV	$\textbf{TP}_{\varvec{ar,}\texttt{w}}$	$\textbf{TP}_{\varvec{ar,}\texttt{s}}$	$\textbf{TP}_{\varvec{ra,}\texttt{w}}$	$\textbf{TP}_{\varvec{ra,}\texttt{s}}$	$\hat{\varvec{\alpha }}$	ABI
IS	1.000	-0.483	-0.419	0.027	0.507	-0.053	0.333	0.323
IV		1.000	0.500	0.012	-0.525	0.111	-0.585	-0.513
$\text {TP}_{ar,\texttt{w}}$			1.000	0.146	-0.475	-0.051	-0.447	-0.367
$\text {TP}_{ar,\texttt{s}}$				1.000	-0.153	-0.371	0.073	0.044
$\text {TP}_{ra,\texttt{w}}$					1.000	0.173	0.300	0.330
$\text {TP}_{ra,\texttt{s}}$						1.000	-0.159	-0.130
$\hat{\alpha }$							1.000	0.789
ABI								1.000

The sensitivity analysis on the impact of the *l* parameter for the DFA-derived metrics is presented in Supplementary material (Section 3). Although both $\hat{\alpha }$ and ABI values for $l=1$ and $l=2$ were highly correlated ($|r| > 0.8$), associations with sociodemographic and health-related factors were more consistent when using $l=1$ than $l=2$.

Figures 4, 5, 6, 7, 8, and 9 show the time series processes of individuals with extreme IS, IV, TP, and DFA values. In footnotes, a short description of what characterized these time series is provided. More figures are available in the Supplementary material (Section 2, Figures S2 to S5).

**Fig. 4:**
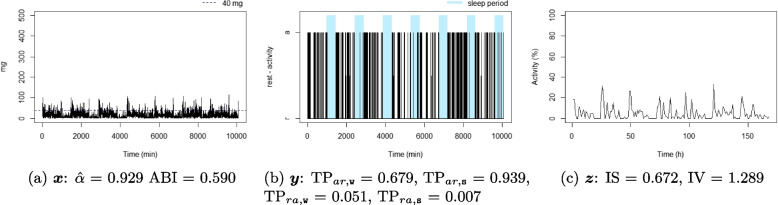
The sedentary: this individual presents the highest $\text {TP}_{ar,\texttt{w}}$. Note that the black blocks in the non-blue region of figure (**b**) are short, ie, this individual has short bouts of activity

**Fig. 5:**
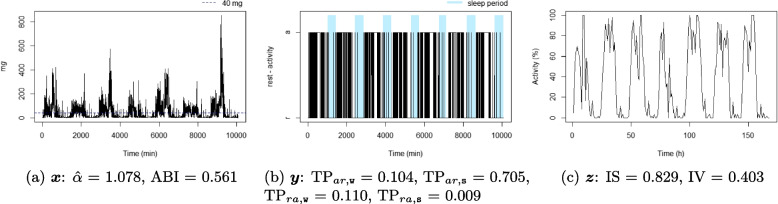
The active: this individual presents the lowest $\text {TP}_{ar,\texttt{w}}$. Note that the black blocks in the non-blue region of figure (**b**) are very long, ie, this individual has long bouts of activity

**Fig. 6:**
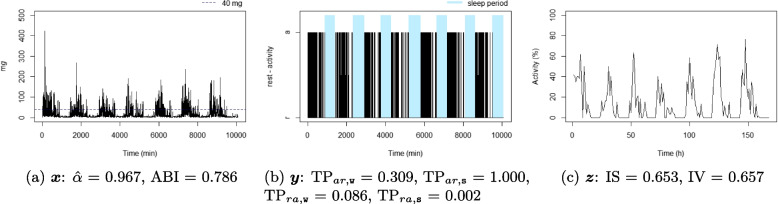
The good sleeper: this individual presents the lowest $\text {TP}_{ra,\texttt{s}}$ and a high $\text {TP}_{ar,\texttt{s}}$. Note that the black (white) blocks in the blue region of figure (**b**) are very brief (long), ie, during the night this individual almost does not display activity

**Fig. 7:**
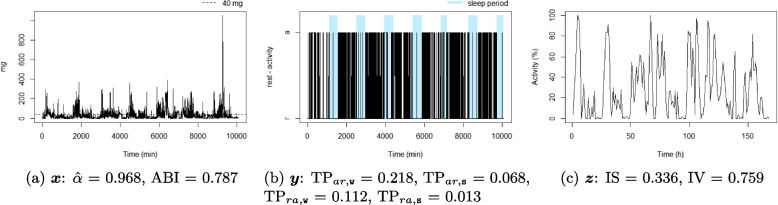
The insomniac: this individual presents the lowest $\text {TP}_{ar,\texttt{s}}$. Note some large black blocks in the blue region of figure (**b**), specially at the third and fifth sleep windows, ie, during the night this individual presents long periods of activity

**Fig. 8:**
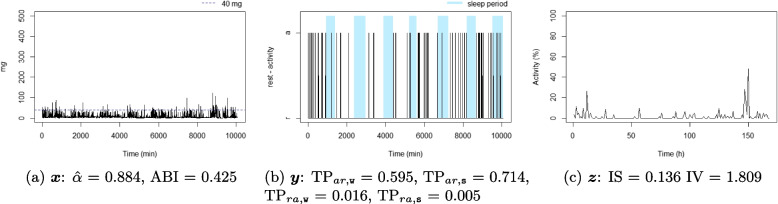
The unbalanced rest-activity person, this individual presents the lowest IS, a high IV, both $\hat{\alpha }$ and ABI low. Note the flat time series in figure (**c**) displays a weak rest-activity pattern and high rhythm fragmentation. Note that the time series in figure (**a**) seems very random (constant spikes without clear difference between day and night), denoting a stationary random noise, ie, low $\hat{\alpha }$ and ABI

**Fig. 9:**
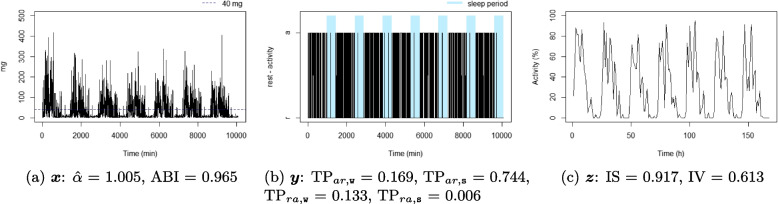
The balanced rest-activity person, this individual presents the highest IS, a low IV and a high ABI. Note the very regular waves in figure (**c**) denoting a high IS and low IV, ie, strong rest-activity pattern and low rhythm fragmentation. Note that the time series in figure (**a**) is very well balanced between smoothness and spikes, display a fractal noise and balanced motion, ie, $\hat{\alpha }$ and ABI close to one

## Discussion

This study provides theoretical ranges and guidance on the interpretation of rest-activity fragmentation metrics. We extended previous research on TP [7, 40] by proposing ML and Bayesian estimators for TP. We also proposed a transformation of DFA-derived self-similarity parameter, the ABI, to reflect the balance of activity behaviours over the observation period. This metric is complementary to the $\alpha$ metric that ought to be used when interest is in characterising the stationarity of the activity pattern. Finally, using accelerometer data from around 2,859 individuals aged 60 to 83 years, we showed that most of the correlations between IS, IV, TP, and ABI were modest. We also found sociodemographic and health-related differences in some of the rest-activity fragmentation metrics but not all, highlighting the fact that they measure different features.

We proposed Bayesian estimators of TP to estimate the probability of change from rest to active period and reversely, defined separately during the awake (day) and the sleep (night) windows. We observed, as expected, a higher TP from activity to rest during the sleep window than during the awake window and, on the reverse, a higher TP from rest to activity during the awake window than during sleep window [41]. We applied these metrics to rest/activity states defined by a threshold of acceleration [17, 18]. These metrics might also be relevant using methods that differentiate sleep and wake states instead of rest and activity states to evaluate the fragmentation of sleep during the night.

When comparing rest-activity fragmentation metrics using data from adults aged 60 to 83 years, we found low to moderate correlations among the variables($|r|<0.6$), except for $\hat{\alpha }$ and ABI ($r=0.789$). Although calculated differently, these estimated correlations are in accordance with those found in the previous studies [7, 25, 26, 42–44]. These modest correlations suggest that these metrics capture distinct features of individuals’ rest-activity patterns. The graphical analysis of the extreme cases of each metric (see Figs. 4, 5, 6, 7, 8, and 9 and Figures S2 to S5 in Section 2 of the Supplementary material) displays several behaviour profiles: sedentary, active, good sleeper, insomniac, (un)balanced rest-activity person, tireless person, and a person with ultradian rhythm. We examined the robustness of our findings regarding the parameter *l* for the DFA-derived metrics ($\alpha$ and ABI) and found that although values for $l=1$ and $l=2$ were highly correlated, associations with sociodemographic and health-related factors were more consistent when using $l=1$, as previously done in other studies [33, 45].

Few studies have examined factors associated with specific rest-activity fragmentation metrics among older adults using data from the Rush Memory and Aging Project [7, 31, 43], the National Health and Nutrition Examination Survey (NHANES) [46, 47], and the Rotterdam study [42]. In these studies, women tended to have higher IS [42, 43, 47] and lower IV [42, 43], higher TP from rest to active state and lower TP from activity to rest [7], while women were found to have a higher $\alpha$ in the NHANES [46] but not in the Rush Memory and Aging Project [31]. Overall, older age was associated with higher IS [42, 43], higher IV [42, 43, 47], higher TPs [7] and lower $\alpha$ [31], although not systematically [47]. In the Rotterdam study, being in employment was associated with lower IS and IV [42] while we found the reverse for IV. Health-related factors such as higher BMI and prevalence of chronic diseases were consistently found associated with lower IS [42, 47, 48], higher IV [42, 47, 48], and higher TP from activity to rest [7], as in the present study. Differences in some of the reported associations might arise from differences in the methods to derive the different metrics and in the sample characteristics. Overall, there is evidence of differences in the rest-activity fragmentation metrics by sociodemographic and health-related factors, supporting future studies to investigate their association with further health outcomes.

The study has several strengths, including the use of both theoretical and empirical demonstrations of the range of the rest-activity fragmentation metrics, using a large sample size. The combination of the approaches increases the validity of our findings. Second, using multiple metrics in the same study population allows for a comprehensive comparison of these metrics. The study has also limitations. We used data from participants who had complete data for seven days. This may have resulted in a selection of the participants, highlighting the need to further investigate the impact of non-wear time on these metrics to allow the use of these metrics in a large sample. In addition, participants were aged between 60 and 83 years and most of them were Caucasian and relatively healthy; whether results are valid in other age and ethnic subgroups requires further investigations. The empirical application is restricted to one type of device, a specific cut-point, 40 m*g*, to differentiate rest from activity, and a specific algorithm to differentiate the sleep from the waking window, and should be replicated in studies using different settings.

## Conclusion

This study provided properties of rest-activity fragmentation metrics previously used and proposed new metrics. Their properties were evaluated using both theorical and empirical approaches among more than 2800 older adults. Overall this study shows that the rest-activity fragmentation metrics examined in this paper - IS, IV, TPs ($\text {TP}_{ra, \texttt{w}}$, $\text {TP}_{ra, \texttt{s}}$, $\text {TP}_{ar, \texttt{w}}$, $\text {TP}_{ar, \texttt{s}}$), $\hat{\alpha }$ and ABI - are modestly correlated, apart for ABI and $\hat{\alpha }$. Additionally, these metrics are differently associated with socio-demographic and health-related factors. Thus, they might reflect different aspects of individual behaviours. However, consideration should be given to their strengths and limitations, as summarized in Table 2. We encourage the use of these metrics in future studies in order to get insight into the role of rest-activity fragmentation for health in complementarity to other circadian rhythm features such as phase and amplitude.

### Supplementary Information

**Supplementary Material 1.**

## Data Availability

Data, protocols, and other metadata of the Whitehall II study are available to the scientific community either via the Whitehall II study data sharing portal (https://www.ucl.ac.uk/psychiatry/research/mental-health-older-people/whitehall-ii/data-sharing). All metrics discussed here were calculated using R software and the codes are available on GitHub (https://github.com/iandanilevicz/frag_metrics), they are available on GGIR ($\ge$3.0-9) R package (https://cran.r-project.org/web/packages/GGIR/index.html), and DFA-$\alpha$ and DFA-ABI are available on DFA ($\ge$1.0-0) R package (https://cran.r-project.org/web/packages/DFA/index.html).

## Abbreviations

ABI: Activity balance index
AR(1): Autoregressive model of order 1
BMI: Body mass index
CI: Confidence interval
CR: Circadian rhythm
DFA: Detrended fluctuation analysis
ENMO: Euclidean norm minus one
IS: Inter-daily stability
IV: Intradaily variability
ML: Maximum likelihood
NHANES: National Health and Nutrition Examination Survey
RAD: Reciprocal average duration
SD: Standard deviation
TP: Transition probability
UK: United Kingdom
