# Supplementary material for: Measures of fragmentation of rest activity patterns: mathematical properties and interpretability based on accelerometer real life data
Source: BMC Med Res Methodol. 2024 Jun 7;24:132. doi: 10.1186/s12874-024-02255-w (PMC11157888; doi:10.1186/s12874-024-02255-w)
Supplement: Supplementary file 1 — Supplementary Material 1. [file 12874_2024_2255_MOESM1_ESM.pdf]

Supplementary material for "Measures of fragmentation of rest activity patterns: mathematical properties and interpretability based on accelerometer real life data"

Ian Meneghel Danilevicz, *Vincent Theodoor van Hees*, *Frank C. T. van der Heide*, *Louis Jacob*, *Benjamin Landré*, *Mohamed Amine Benadjaoud and Séverine Sabia*

Abstract

This report contains supplemental material for the paper entitled "Measures of fragmentation of rest activity patterns: mathematical properties and interpretability based on accelerometer real life data", hereinafter referred to as the "Manuscript". Section 1 contains the mathematical proofs of all the theorems presented in the Manuscript. Section 2 reports additional figures from the real data analyses; precisely, it displays additional time series processes with extreme metrics complementary to the ones presented in the Manuscript. Section 3 presents results from a sensitivity analysis for DFA, where we compare two different values for a tuning parameter, l, that is used for the calculation of α and ABI.

Keywords: Circadian rhythm, detrended fluctuation analysis, inter-daily stability, intradaily variability, transition probability, Whitehall II cohort.

1 Mathematical proofs

Here we present the mathematical proofs of Theorems 1 to 4 introduced in the Manuscript. The Corollary 5 is also proofed here, but Corollary 6 was already proofed in the Manuscript, as it is a direct consequence of Corollary 5. The numbering of the equations is not reset, as we need to eventually mention equations from the Manuscript, by this way we avoid ambiguity.

Theorem 1

Given a stochastic process $\{Z_p\}_{p\in P}$, $\text{IS}(\boldsymbol{z}) \in [0,1]$.

Proof. The inter-daily stability (IS) denominator is

$$\begin{aligned}\sum_{h=1}^{H}\sum_{d=1}^{D}(z_{d,h}-\bar{z})^2 &= \sum_{h=1}^{H}\sum_{d=1}^{D}(z_{d,h}-\bar{z}_h+\bar{z}_h-\bar{z})^2\\ &= \sum_{h=1}^{H}\sum_{d=1}^{D}(z_{d,h}-\bar{z}_h)^2+\sum_{h=1}^{H}\sum_{d=1}^{D}(\bar{z}_h-\bar{z})^2+2\sum_{h=1}^{H}\sum_{d=1}^{D}(z_{d,h}-\bar{z}_h)(\bar{z}_h-\bar{z})\\ &= \sum_{h=1}^{H}\sum_{d=1}^{D}(z_{d,h}-\bar{z}_h)^2+D\sum_{h=1}^{H}(\bar{z}_h-\bar{z})^2+2\sum_{h=1}^{H}(\bar{z}_h-\bar{z})\sum_{d=1}^{D}(z_{d,h}-\bar{z}_h) \qquad (1)\\ &= \sum_{h=1}^{H}\sum_{d=1}^{D}(z_{d,h}-\bar{z}_h)^2+D\sum_{h=1}^{H}(\bar{z}_h-\bar{z})^2 \geq 0, \qquad (2)\end{aligned}$$

where the third term of (1) is zero by the definition of $\bar{z}_h$. The numerator of IS is $D\sum_{h=1}^{H}(\bar{z}_h-\bar{z})^2$, which appears as a part of the denominator in (2), consequently $\text{IS}(\boldsymbol{z}) \leq 1$.

Thus

$$\text{IS}(\boldsymbol{z}) = \frac{\sum_{h=1}^{H}(\bar{z}_h-\bar{z})^2}{D^{-1}\sum_{h=1}^{H}\sum_{d=1}^{D}(z_{d,h}-\bar{z}_h)^2+\sum_{h=1}^{H}(\bar{z}_h-\bar{z})^2},$$

so that a higher value of IS reflects a higher proportion of the overall variance ($D^{-1}\sum_{h=1}^{H}\sum_{d=1}^{D}(z_{d,h}-\bar{z}_h)^2 + \sum_{h=1}^{H}(\bar{z}_h-\bar{z})^2$) being explained by the variance between the average hours of the day ($\sum_{h=1}^{H}(\bar{z}_h-\bar{z})^2$).

□

Theorem 2

Given a stochastic process $\{Z_p\}_{p\in P}$ and under assumption (A1), $\mathrm{IV}(\boldsymbol{z}) \in [0,\infty)$.

Proof. The intradaily variability (IV) numerator's core is given by

$$
\begin{aligned}
\sum_{p=2}^{P}(Z_p - Z_{p-1})^2 &= \sum_{p=2}^{P} Z_p^2 - 2\sum_{p=2}^{P} Z_p Z_{p-1} + \sum_{p=2}^{P} Z_{p-1}^2 && (3)\\
&= \sum_{p=2}^{P} Z_p^2 - 2\sum_{p=2}^{P}(\mu + \phi Z_{p-1} + \epsilon_p) Z_{p-1} + \sum_{p=2}^{P} Z_{p-1}^2 && (4)\\
&= \sum_{p=2}^{P} Z_p^2 - 2\mu\sum_{p=2}^{P} Z_{p-1} - 2\phi\sum_{p=2}^{P} Z_{p-1}^2 - 2\sum_{p=2}^{P}(Z_{p-1}\epsilon_p) + \sum_{p=2}^{P} Z_{p-1}^2\\
&= \sum_{p=2}^{P} Z_p^2 - 2\mu\sum_{p=1}^{P-1} Z_p - 2\phi\sum_{p=1}^{P-1} Z_p^2 - 2\sum_{p=1}^{P-1}(Z_p\epsilon_{p+1}) + \sum_{p=1}^{P-1} Z_p^2\\
&= \sum_{p=2}^{P} Z_p^2 - 2\phi\sum_{p=1}^{P-1} Z_p^2 + \sum_{p=1}^{P-1} Z_p^2 - 2\mu\sum_{p=1}^{P-1} Z_p - 2\sum_{p=1}^{P-1}(Z_p\epsilon_{p+1})\\
&= \sum_{p=1}^{P} Z_p^2 - Z_1^2 - 2\phi\sum_{p=1}^{P} Z_p^2 + 2\phi Z_P^2 + \sum_{p=1}^{P} Z_p^2 - Z_P^2 - 2\mu\sum_{p=1}^{P} Z_p + 2\mu Z_P - 2\sum_{p=1}^{P-1}(Z_p\epsilon_{p+1})\\
&= 2(1-\phi)\sum_{p=1}^{P} Z_p^2 - Z_1^2 + 2\phi Z_P^2 - Z_P^2 - 2\mu P\bar{z} + 2\mu Z_P - 2\sum_{p=1}^{P-1}(Z_p\epsilon_{p+1})\\
&= 2(1-\phi)P\sigma^2 - 2\mu P\bar{z} - 2\eta + \delta\\
&= 2P\left[(1-\phi)\sigma^2 - \mu\bar{z} - \frac{\eta}{P} + \frac{\delta}{2P}\right]\\
&= 2P\,[\omega] \geq 0, && (5)
\end{aligned}
$$

where $\sigma^2 = P^{-1}\sum_{p=1}^{P} Z_p^2$, $\delta = -Z_1^2 + 2\phi Z_P^2 - Z_P^2 + 2\mu Z_P$, $\eta = \sum_{p=1}^{P-1}(Z_p\epsilon_{p+1})$, and $\omega = (1-\phi)\sigma^2 - \mu\bar{z} - \frac{\eta}{P} + \frac{\delta}{2P}$. The replacing operation of values from line (3) to (4) is allowed by assumption (A1). The inequality (5) guaranties that $\omega \geq 0$. The IV denominator's core is given by

$$\sum_{p=1}^{P}(Z_p - \bar{z})^2 = P(\sigma^2 - \bar{z}^2) > 0, \tag{6}$$

where the equality to zero is not considered in (6) for existence of IV. The (6) guaranties that $\sigma^2 > \bar{z}^2$ (eg Jensen's inequality). The IV as defined in (1) from Manuscript is equal to

$$\mathrm{IV}(\boldsymbol{z}) = \frac{P\sum_{p=2}^{P}(Z_p - Z_{p-1})^2}{(P-1)\sum_{p=1}^{P}(Z_p - \bar{z})^2} = \frac{2P^2\left[(1-\phi)\sigma^2 - \mu\bar{z} - \frac{\eta}{P} + \frac{\delta}{2P}\right]}{(P-1)(P(\sigma^2 - \bar{z}^2))} = \frac{2P\left[(1-\phi)\sigma^2 - \mu\bar{z} - \frac{\eta}{P} + \frac{\delta}{2P}\right]}{(P-1)(\sigma^2 - \bar{z}^2)}. \tag{7}$$

The value of IV depends of P, ϕ, σ^2, μ, $\bar{z}$, Z_1, Z_P, and $\sum_{p=1}^{P-1}(Z_p\epsilon_{p+1})$. However, two special situations are highlighted. If $\omega = 0$, then $\mathrm{IV}(\boldsymbol{z}) = 0$, and if $\omega > 0$ and $\bar{z}^2 \to \sigma^2$, then

$$\lim_{\bar{z}^2\to\sigma^2} \mathrm{IV}(\boldsymbol{z}) = \lim_{\bar{z}^2\to\sigma^2} \frac{2P\left[(1-\phi)\sigma^2 - \mu\bar{z} - \frac{\eta}{P} + \frac{\delta}{2P}\right]}{(P-1)(\sigma^2 - \bar{z}^2)} = \lim_{\Delta\to 0}\frac{2P\,[\omega]}{(P-1)\Delta} = \infty, \tag{8}$$

for any $P \geq 2$. Note that (8) is positive as $\omega > 0$ and $\Delta > 0$ because $\sigma^2 > \bar{z}^2$. □

Theorem 3

Given a stochastic process $\{Z_p\}_{p \in P}$ and under assumptions (A1) and (A2), $\lim_{P\to\infty}(\mathrm{IV}(\boldsymbol{z})) \in [0,2]$.

Proof. Given (A1) the Theorem 2's condition is given and we may reorganize equation (7) as

$$\mathrm{IV}(\boldsymbol{z}) = \frac{2P\left[(1-\phi)\sigma^2 - \mu\bar{z}\right]}{(P-1)(\sigma^2-\bar{z}^2)} + \frac{\delta}{(P-1)(\sigma^2-\bar{z}^2)} - \frac{2\eta}{(P-1)(\sigma^2-\bar{z}^2)}, \tag{9}$$

the asymptotic behaviour of each part of (9) is treated separately in the following three parts:

Part 1) By law of large numbers $\lim_{P\to\infty} \bar{z} = \mu$ and by continuous mapping theorem

$$\lim_{P\to\infty}\left[\frac{2P\left[(1-\phi)\sigma^2 - \mu\bar{z}\right]}{(P-1)(\sigma^2-\bar{z}^2)}\right] = \frac{2\left[(1-\phi)\sigma^2-\mu^2\right]}{(\sigma^2-\mu^2)}.$$

Part 2) As an autoregressive model, Z_p may be rewritten as

$$Z_p = \frac{1-\phi^2}{1-\phi}\mu + \sum_{k=1}^{P-1}\phi^{p-k}\epsilon_k,$$

where $\epsilon_k \sim \mathcal{N}(0,\sigma_\varepsilon^2)\,\forall k$. So by construction, Z_p is a Gaussian random variable with known expectations $\mu_p = \frac{1-\phi^p}{1-\phi}\mu$ and known variance $\sigma_p^2 = \frac{1-\phi^{2p}}{1-\phi^2}\sigma_\varepsilon^2$. We also have $\mathbb{E}(Z_p^2) = \sigma_p^2 + \mu_p^2$ and $\mathbb{V}(Z_p^2) = \mathbb{E}(Z_p^4) - \mathbb{E}(Z_p^2)^2 = 3\sigma_p^4 + 6\sigma_p^2\mu_p^2 + \mu_p^4 - (\sigma_p^2+\mu_p^2)^2$. As $|\phi| < 1$, we observe $\lim_{P\to\infty}\mu_p = \frac{\mu}{1-\phi}$ and $\lim_{P\to\infty}\sigma_p^2 = \frac{\sigma_\varepsilon^2}{1-\phi^2}$, then $\lim_{P\to\infty}\mathbb{E}\left(\frac{Z_p}{P}\right) = 0$ and $\lim_{P\to\infty}\mathbb{V}\left(\frac{Z_p}{P}\right) = 0$, which implies that $\lim_{P\to\infty}\left(\frac{Z_p}{P}\right) = 0$ almost sure (a.s.) (by Tchebychev inequality for example). And also $\lim_{P\to\infty}\mathbb{E}\left(\frac{Z_p^2}{P}\right) = 0$ and $\lim_{P\to\infty}\mathbb{V}\left(\frac{Z_p^2}{P}\right) = 0$, which implies that $\lim_{P\to\infty}\left(\frac{Z_p^2}{P}\right) = 0$ a.s.. As the previous result is for any Z_p and Z_p^2, it is true for Z_P, Z_P^2 and Z_1^2, concluding

$$\lim_{P\to\infty}\frac{\delta}{(P-1)(\sigma^2-\bar{z}^2)} = \lim_{P\to\infty}\frac{2\phi Z_P^2 - Z_P^2 + 2\mu Z_P - Z_1^2}{(P-1)(\sigma^2-\bar{z}^2)} = 0.$$

Part 3) Remember that $\eta = \sum_{p=1}^{P-1}(z_p\epsilon_{p+1})$, and we cannot use the law of large numbers based approach since the random variables Z_p are not independent and not identically distributed. Note that the random vectors $(Z_1,\ldots,Z_{P-1})'$ and $(\epsilon_1,\ldots,\epsilon_{P-1})'$ are related by the following matrix relationship

$$\begin{pmatrix} Z_1 \\ \vdots \\ Z_{P-1}\end{pmatrix} = \begin{pmatrix} \mu_1 \\ \vdots \\ \mu_{P-1}\end{pmatrix} + \Pi_{P-1}\begin{pmatrix} \epsilon_1 \\ \vdots \\ \epsilon_{P-1}\end{pmatrix}, \text{ where } \Pi_{P-1} = \begin{pmatrix} 1 & \cdots & 0 \\ \vdots & \ddots & \vdots \\ \phi^{P-2} & \cdots & 1\end{pmatrix}$$

is a lower triangular matrix of dimension $P-1$ and each element of the diagonal equal to one. Thus, η

can be written as follows

$$
\begin{aligned}
\eta &= (\epsilon_2, \cdots, \epsilon_P)(Z_1, \cdots, Z_{P-1})' \\
&= (\epsilon_2, \cdots, \epsilon_P) \left[\begin{pmatrix} \mu_1 \\ \vdots \\ \mu_{P-1} \end{pmatrix} + \Pi_{P-1} \begin{pmatrix} \epsilon_1 \\ \vdots \\ \epsilon_{P-1} \end{pmatrix} \right] \\
&= (\epsilon_2, \cdots, \epsilon_P) \begin{pmatrix} \mu_1 \\ \vdots \\ \mu_{P-1} \end{pmatrix} + (\epsilon_2, \cdots, \epsilon_P) \Pi_{P-1} \begin{pmatrix} \epsilon_1 \\ \vdots \\ \epsilon_{P-1} \end{pmatrix} \\
&= (\epsilon_2, \cdots, \epsilon_P)(\mu_1, \cdots, \mu_{P-1})' + \boldsymbol{\epsilon}' \begin{pmatrix} \mathbf{0}'_{P-1} \\ \mathrm{I}_{P-1} \end{pmatrix} \Pi_{P-1} (\mathrm{I}_{P-1}, \mathbf{0}_{P-1}) \boldsymbol{\epsilon} \\
&= (\epsilon_2, \cdots, \epsilon_P)(\mu_1, \cdots, \mu_{P-1})' + \boldsymbol{\epsilon}' \tilde{\Pi}_P \boldsymbol{\epsilon} \\
&= \eta_1 + \eta_2,
\end{aligned}
$$

where $\boldsymbol{\epsilon} = (\epsilon_1, \ldots, \epsilon_P)'$, I_{P-1} is the identity matrix of dimension $P-1$, $\mathbf{0}_{P-1}$ is the vector of zeros with length equal to $P-1$, $\tilde{\Pi}_P$ is a matrix of dimension P resulting from $(\mathbf{0}'_{P-1}, \mathrm{I}_{P-1})' \Pi_{P-1} (\mathrm{I}_{P-1}, \mathbf{0}_{P-1})$. We call $\eta_1 = (\epsilon_2, \cdots, \epsilon_P)(\mu_1, \cdots, \mu_{P-1})'$ and $\eta_2 = \boldsymbol{\epsilon}' \tilde{\Pi}_P \boldsymbol{\epsilon}$ and treat the asymptotic convergence of each part in a separate item.

Part 3.1) On one hand, we have a Gaussian random variable as $\eta_1 = (\epsilon_2, \cdots, \epsilon_P)(\mu_1, \cdots, \mu_{P-1})' = \sum_{p=1}^{P-1} \mu_p \epsilon_{p+1} \sim \mathcal{N}(0, \sum_{p=1}^{P-1} \mu_p^2)$. Then $\mathbb{E}(\eta_1) = 0$ and

$$
\begin{aligned}
\mathbb{V}(\eta_1) &= \sum_{p=1}^{P-1} \mu_p^2 \\
&= \left(\frac{\mu}{1-\phi} \right)^2 \sum_{p=1}^{P-1} (1 - \phi^p)^2 \\
&\leq \left(\frac{\mu}{1-\phi} \right)^2 \sum_{p=1}^{P-1} (1 - \phi^p) \qquad (10) \\
&\leq \left(\frac{\mu}{1-\phi} \right)^2 \left(P - \frac{1-\phi^P}{1-\phi} \right),
\end{aligned}
$$

where the inequality in (10) is guaranteed since $0 < \phi < 1$ given by condition (A2). Consequently, $\lim_{P\to\infty} \mathbb{V}(\frac{\eta_1}{P-1}) = \lim_{P\to\infty} \frac{1}{(P-1)^2} \mathbb{V}(\eta_1) = 0$, which ensures that $\lim_{P\to\infty} (\frac{\eta_1}{P-1}) = 0$ a.s..

Part 3.2) On the other, hand we have a Gaussian quadratic form as $\eta_2 = \boldsymbol{\epsilon}' \tilde{\Pi}_P \boldsymbol{\epsilon}$. We have by the law of large numbers that $\lim_{P\to\infty} \frac{\sum_{p=1}^{P} \epsilon_p^2}{P} = \mathbb{E}(\epsilon_1^2) = \sigma_\varepsilon^2$, so the expectation of η_2 follows

$$
\mathbb{E}(\boldsymbol{\epsilon}' \tilde{\Pi}_P \boldsymbol{\epsilon}) = \sigma_\varepsilon^2 \, \mathrm{Tr}(\tilde{\Pi}_P) = 0,
$$

as each element of the diagonal of $\tilde{\Pi}_P$ is zero. The variance of η_2 follows

$$\begin{aligned}
\mathbb{V}(\boldsymbol{\epsilon}'\tilde{\Pi}_P\boldsymbol{\epsilon}) &= \mathbb{E}((\boldsymbol{\epsilon}'\tilde{\Pi}_P\boldsymbol{\epsilon})^2) - (\mathbb{E}(\boldsymbol{\epsilon}'\tilde{\Pi}_P\boldsymbol{\epsilon}))^2 \\
&= \mathbb{E}((\boldsymbol{\epsilon}'\tilde{\Pi}_P\boldsymbol{\epsilon})^2) \\
&= \sigma_\varepsilon^4 \operatorname{Tr}(\tilde{\Pi}_P^2) \\
&\leq \sigma_\varepsilon^4 \operatorname{Tr}(\tilde{\Pi}_P'\tilde{\Pi}_P) \qquad (11) \\
&\leq \sigma_\varepsilon^4 \operatorname{Tr}\left(\begin{pmatrix} \mathbf{0}_{P-1} & \Pi_{P-1}' \\ 0 & \mathbf{0}_{P-1}' \end{pmatrix}\begin{pmatrix} \mathbf{0}_{P-1}' & 0 \\ \Pi_{P-1} & \mathbf{0}_{P-1} \end{pmatrix}\right) \\
&\leq \sigma_\varepsilon^4 \operatorname{Tr}\left(\begin{pmatrix} \Pi_{P-1}'\Pi_{P-1} & \mathbf{0}_{P-1} \\ \mathbf{0}_{P-1}' & 0 \end{pmatrix}\right) \\
&\leq \sigma_\varepsilon^4 \operatorname{Tr}\left(\Pi_{P-1}'\Pi_{P-1}\right), \qquad (12)
\end{aligned}$$

where the inequality in (11) is a consequence of eigenvalues and singular values inequality. For a given $1 \leq i \leq P-1$:

$$(\Pi_{P-1}'\Pi_{P-1})_{ii} = \sum_{p=1}^{P-1}(\Pi_{P-1}')_{ip}(\Pi_{P-1})_{pi} = \sum_{p=1}^{i}(\Pi_{P-1}')_{pi}^2 = \sum_{p=1}^{i}\phi^{(2(i-p)} = \frac{1-\phi^{2i}}{1-\phi^2}, \qquad (13)$$

where the last equality is the sum of the first i terms of a geometric series. Let us continue the variance expression from line (12) using the result from (13) as follows

$$\begin{aligned}
\mathbb{V}(\boldsymbol{\epsilon}'\tilde{\Pi}_P\boldsymbol{\epsilon}) &\leq \sigma_\varepsilon^4 \operatorname{Tr}\left(\Pi_{P-1}'\Pi_{P-1}\right) \\
&\leq \sigma_\varepsilon^4 \sum_{p=1}^{P-1}\frac{1-\phi^{2p}}{1-\phi^2} \\
&\leq \frac{\sigma_\varepsilon^4}{1-\phi^2}\sum_{p=1}^{P-1} 1-\phi^{2p} \\
&\leq \frac{\sigma_\varepsilon^4 P}{1-\phi^2}, \qquad (14)
\end{aligned}$$

where (14) is a strict inequality for any $\phi > 0$. We conclude that $\lim_{P\to\infty}\left(\mathbb{V}(\frac{\boldsymbol{\epsilon}'\tilde{\Pi}_P\boldsymbol{\epsilon}}{P-1})\right) = \lim_{P\to\infty}\left(\frac{\mathbb{V}(\boldsymbol{\epsilon}'\tilde{\Pi}_P\boldsymbol{\epsilon})}{(P-1)^2}\right) = 0$ and, consequently $\lim_{P\to\infty}\left(\frac{\eta_2}{P-1}\right) = 0$ a.s..

By parts 3.1 and 3.2 we have that $\lim_{P\to\infty}\left(\frac{\eta}{(P-1)(\sigma^2-\bar{z}^2)}\right) = \lim_{P\to\infty}\left(\frac{\eta_1+\eta_2}{(P-1)(\sigma^2-\bar{z}^2)}\right) = 0$ a.s.. By parts 1, 2 and 3 we have that

$$\lim_{P\to\infty} \operatorname{IV}(\boldsymbol{z}) = \frac{2\left[(1-\phi)\sigma^2-\mu^2\right]}{(\sigma^2-\mu^2)},$$

for which the maximum equal to 2 is achieved at $\phi = 0$ under the restriction imposed by (A2).

□

Theorem 4

Given a stochastic process $\{Y_t\}_{t\in T}$, under assumptions (B1) and (B2), the maximum likelihood (ML) estimator of $\pi_{ra}(s)$ and $\pi_{ar}(s)$ are $\hat{\pi}_{ra}(s)_{ML} = \frac{\sum_{i=1}^{n_r} I(r_i\geq s)-I(y_T=r)}{\sum_{i=1}^{n_r}(r_i-s+1)I(r_i\geq s)-I(y_T=r)}$ and $\hat{\pi}_{ar}(s)_{ML} = \frac{\sum_{i=1}^{n_a} I(a_i\geq s)-I(y_T=a)}{\sum_{i=1}^{n_a}(a_i-s+1)I(a_i\geq s)-I(y_T=a)}$, for $s = 1,\dots,S_r-1$, and $s = 1,\dots,S_a-1$, respectively.

Proof. Under assumption (B2), given $Y_{t-1} = r,\dots,Y_{t-s} = r$, then $Y_t \sim \mathcal{B}(\pi_{ra}(s,t))$, where $\mathcal{B}(\pi_{ra}(s,t))$ is a Bernoulli distribution with probability of "success", eg, transition from r to a, equal to $\pi_{ra}(s,t)$. Assumption

(B2) ensures that given s steps backward, the transition probability can be well defined as a Bernoulli because the process is dual. Given assumption (B1) the probability $\pi_{ra}(s,t)$ is constant to any t, let us say $\pi_{ra}(s)$. This process is different from the one studied by Anderson and Goodman (1957) [1], but the events are still countable, and following the same lines, the ML estimator of $\pi_{ra}(s)$ is given by the ratio between the sum of "success" cases and the sum of trials as

$$\begin{aligned}\hat{\pi}_{ra}(s)_{ML} &= \frac{\sum_{t=1}^{T-s} I(y_t = r, \ldots, y_{t+s-1} = r, y_{t+s} = a)}{\sum_{t=1}^{T-s} I(y_t = r, \ldots, y_{t+s-1} = r)} \\ &= \frac{\sum_{i=1}^{n_r} I(r_i \geq s)}{\sum_{i=1}^{n_r} I(r_i \geq s) + \sum_{i=1}^{n_r} (r_i - s) I(r_i \geq s)} \\ &= \frac{\sum_{i=1}^{n_r} I(r_i \geq s)}{\sum_{i=1}^{n_r} (r_i - s + 1) I(r_i \geq s)}, \end{aligned} \tag{15}$$

if $y_T = a$, then after any bout of rest there is activity. Consequently, the number of *successes* (numerator) equals the number of bouts of rest with length $\geq s$, and the number of *trials* (denominator) equals the number of intervals of rest with length equal to s. For example, for $s = 4$, a bout of rest with length equal to four ($r_i = 4$) counts one time for the numerator and one time for the denominator because there is one interval with a length of four in this bout; a bout of rest with length equal to five counts once for the numerator and twice for the denominator as there are two intervals with a length of four in that bout. We need to repeat this operation for all $r_i \geq s$.

However, if $y_T = r$, then we cannot count the last event, because we do not know how to classify it. Then

$$\hat{\pi}_{ra}(s)_{ML} = \frac{\sum_{i=1}^{n_r} I(r_i \geq s) - 1}{\sum_{i=1}^{n_r} (r_i - s + 1) I(r_i \geq s) - 1}, \tag{16}$$

if $y_T = r$. The combination of both situations (15) and (16) gives

$$\hat{\pi}_{ra}(s)_{ML} = \frac{\sum_{i=1}^{n_r} I(r_i \geq s) - I(y_T = r)}{\sum_{i=1}^{n_r} (r_i - s + 1) I(r_i \geq s) - I(y_T = r)}, \tag{17}$$

for any value of y_T. In a symmetric way,

$$\hat{\pi}_{ar}(s)_{ML} = \frac{\sum_{i=1}^{n_a} I(a_i \geq s) - I(y_T = a)}{\sum_{i=1}^{n_a} (a_i - s + 1) I(a_i \geq s) - I(y_T = a)}.$$

□

Corollary 5

Given a stochastic process $\{Y_t\}_{t \in T}$, under assumptions (B1) and (B2), the ML estimators of $\pi_{ra}(1)$ and $\pi_{ar}(1)$ are $\hat{\pi}_{ra}(1)_{ML} = \frac{n_r - I(y_T = r)}{T_r - I(y_T = r)}$ and $\hat{\pi}_{ar}(1)_{ML} = \frac{n_a - I(y_T = a)}{T_a - I(y_T = a)}$.

Proof. This is a special case of Markov chain for dichotomous outcome, Anderson and Goodman (1957) demonstrated the ML estimator for a Markov chain with multiple outcomes in equation (2.8) [1]. Following the same lines, the ML estimator of $\pi_{ra}(1)$ is given by the ratio between the sum of "success" cases and the sum of trials, just replace $s = 1$ in (17) as

$$\hat{\pi}_{ra}(1)_{ML} = \frac{\sum_{i=1}^{n_r} I(r_i \geq 1) - I(y_T = r)}{\sum_{i=1}^{n_r} (r_i) I(r_i \geq 1) - I(y_T = r)} = \frac{n_r - I(y_T = r)}{T_r - I(y_T = r)}.$$

By symmetry, the ML estimator of $\pi_{ar}(1)$ is given by

$$\hat{\pi}_{ar}(1)_{ML} = \frac{n_a - I(y_T = a)}{T_a - I(y_T = a)}.$$

□

2 Additional results

Figure S1 displays the mean predicted values for each metric by the age range observed in the study sample (from 60 to 83 years) using coefficients obtained in Table 4 of the Manuscript (non standardized). This figure is useful to interpret the quadratic effect of the age. Figures S2 to S5 show the time series processes of individuals with extreme IS, IV, TP, and DFA values not displayed in the Manuscript. In footnotes, a short description of what characterized these time series is provided. Eventhough, the correspondence between the values of the metrics and the shape of the time series is intuitive after the argument presented in the Manuscript, these figures provide completeness to the interpretation of the proposed metrics.

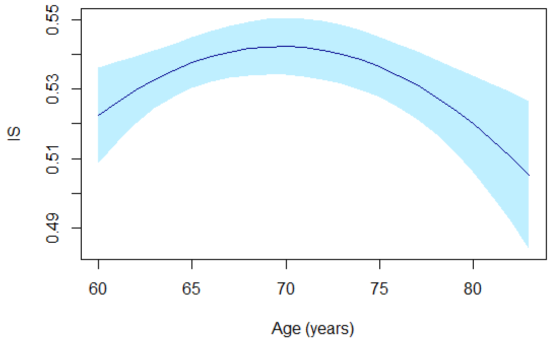

(a) IS

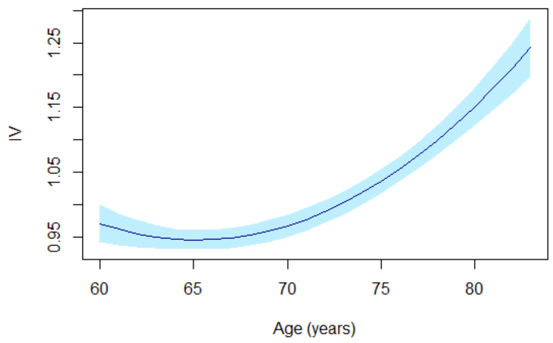

(b) IV

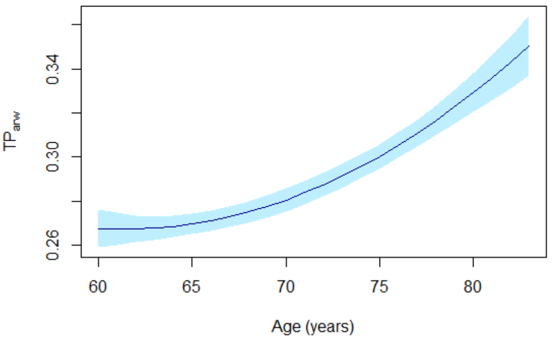

(c) $\mathrm{TP}_{ar,\mathtt{w}}$

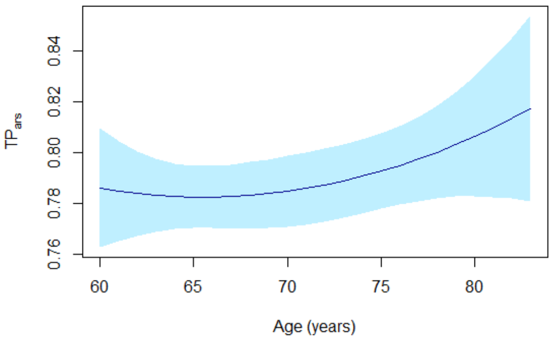

(d) $\mathrm{TP}_{ar,\mathtt{s}}$

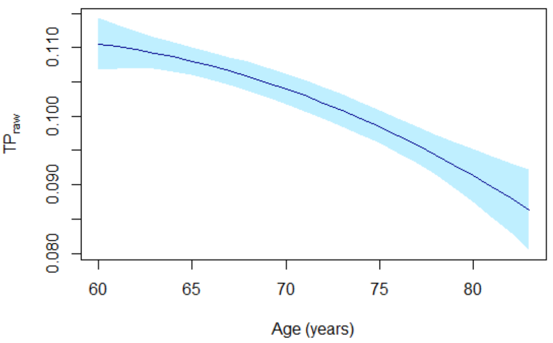

(e) $\mathrm{TP}_{ra,\mathtt{w}}$

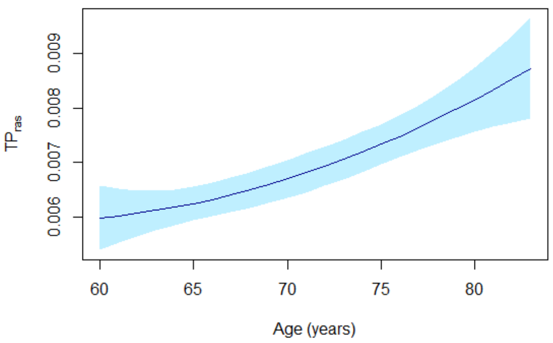

(f) $\mathrm{TP}_{ra,\mathtt{s}}$

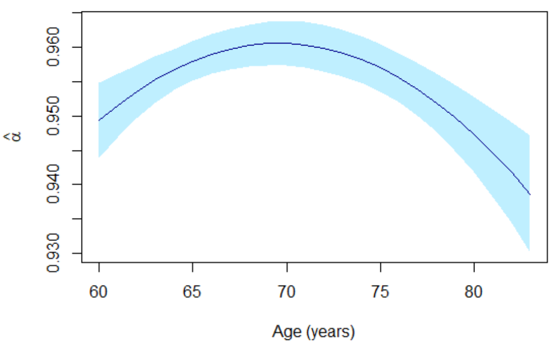

(g) $\hat{\alpha}$

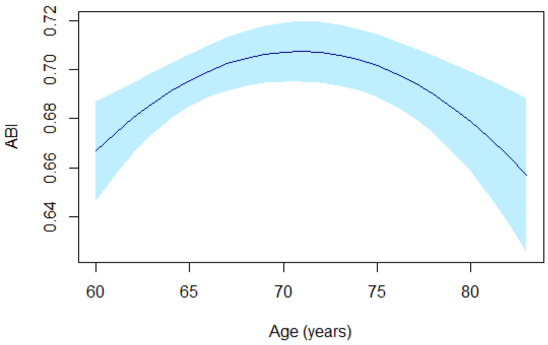

(h) ABI

Figure S1: Mean predicted values for each metric by the age range observed in the study population (from 60 to 83 years) using coefficients obtained in Table 4 of the Manuscript (non standardized values).

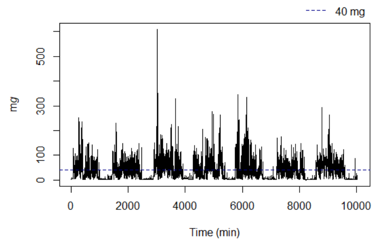

(a) $\boldsymbol{x}$: $\hat{\alpha}$ = 1.011, ABI = 0.923

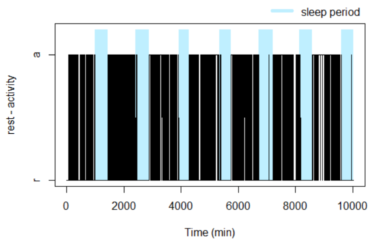

(b) $\boldsymbol{y}$: $\text{TP}_{ar,\mathtt{w}}$ = 0.170, $\text{TP}_{ar,\mathtt{s}}$ = 0.610, $\text{TP}_{ra,\mathtt{w}}$ = 0.262, $\text{TP}_{ra,\mathtt{s}}$ = 0.006

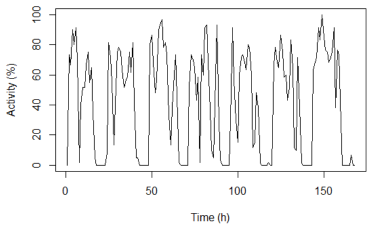

(c) $\boldsymbol{z}$: IS = 0.717, IV = 0.586

Figure S2: The tireless person: this individual presents the highest $\tilde{\pi}_{ra,\mathtt{w}}$. Note that the white blocks in the non-blue region of figure (b) are very short, ie, this individual has short bouts of rest.

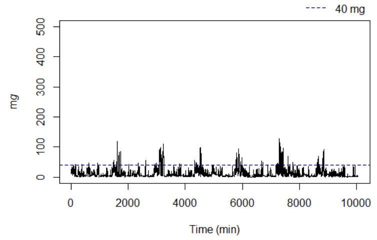

(a) $\boldsymbol{x}$: $\hat{\alpha}$ = 1.030, ABI = 0.801

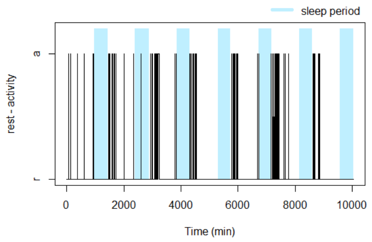

(b) $\boldsymbol{y}$: $\text{TP}_{ar,\mathtt{w}}$ = 0.219, $\text{TP}_{ar,\mathtt{s}}$ = 1.000, $\text{TP}_{ra,\mathtt{w}}$ = 0.014, $\text{TP}_{ra,\mathtt{s}}$ = 0.003

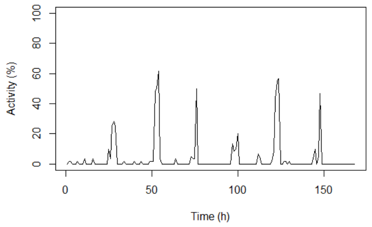

(c) $\boldsymbol{z}$: IS = 0.460, IV = 0.960

Figure S3: The most sedentary: this individual presents the lowest $\tilde{\pi}_{ra,\mathtt{w}}$. Note that the white blocks in the non-blue region of figure (b) are very large, ie, this individual has long bouts of rest.

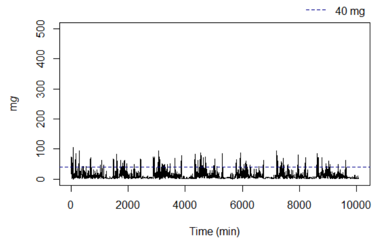

(a) $\boldsymbol{x}$: $\hat{\alpha}$ = 0.915, ABI = 0.532

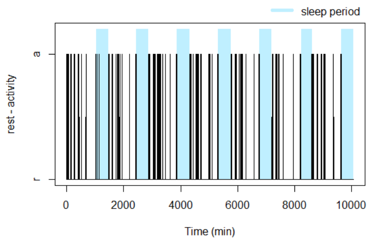

(b) $\boldsymbol{y}$: $\text{TP}_{ar,\mathtt{w}}$ = 0.567, $\text{TP}_{ar,\mathtt{s}}$ = 0.789, $\text{TP}_{ra,\mathtt{w}}$ = 0.032, $\text{TP}_{ra,\mathtt{s}}$ = 0.005

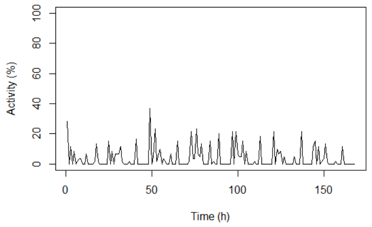

(c) $\boldsymbol{z}$: IS = 0.683, IV = 2.117

Figure S4: The person with an ultradian rhythm: this individual presents the highest IV and the highest $\tilde{\pi}_{ra,\mathtt{s}}$. Note that the time series in figure (c) presents high frequency, so that waves of activity are shorter than one day; this characterizes an ultradian rhythm. This is coherent with a negative autocorrelation parameter $\hat{\phi}$ = -0.100.

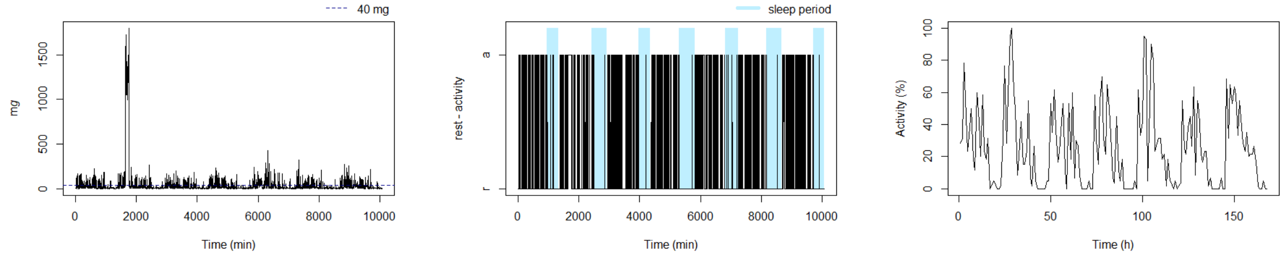

(a) $\boldsymbol{x}$: $\hat{\alpha} = 1.223$, ABI $= 0.193$

(b) $\boldsymbol{y}$: $\mathrm{TP}_{ar,\mathtt{w}} = 0.193$, $\mathrm{TP}_{ar,\mathtt{s}} = 0.477$, $\mathrm{TP}_{ra,\mathtt{w}} = 0.109$, $\mathrm{TP}_{ra,\mathtt{s}} = 0.009$

(c) $\boldsymbol{z}$: IS $= 0.574$, IV $= 1.016$

Figure S5: The unbalanced rest-activity person, this individual presents the highest $\hat{\alpha}$, the lowest ABI, intermediate levels of both IS and IV. Note that the time series in figure (a) presents an isolated high peak, which breaks the stationarity of the process and causes a high $\hat{\alpha}$ and low ABI. Of course, this does not affect figure (c) which is a proportion, consequently IS and IV are not affected, reflecting an average pattern.

3 Sensitivity analysis for DFA

Here, findings for α and ABI using a different polynomial order for equation (3) of the Manuscript ($l = 1$ and $l = 2$) are compared. We found that women present significantly higher average values of $\hat{\alpha}$(with $l = 1$) and ABI(with $l = 1, 2$) and no significant difference was observed between age ≥ 70 vs age < 70 for $\hat{\alpha}$ and ABI using either $l = 1$ or $l = 2$, see Table S1. Table S2 shows that both $\hat{\alpha}(l = 1)$ and ABI($l = 1$) were associated with all socio-demographic (except education) and health-related factors. However, $\hat{\alpha}(l = 2)$ was not significantly associated with sex and education, and ABI($l = 2$) was not associated with employment status, education, and morbidities. Finally, the Pearson's correlations were 0.931 between $\hat{\alpha}(l = 1)$ and $\hat{\alpha}(l = 2)$, and 0.878 between ABI($l = 1$) and ABI($l = 2$).

Table S1: Mean (SD) of estimated self-similarity parameter ($\hat{\alpha}$) and activity balance index (ABI) using $l = 1, 2$.

	all	men	women		age < 70	age ≥ 70	
N	2859	2257	602		1717	1142	
	mean (SD)	mean (SD)	mean (SD)	p-value	mean (SD)	mean (SD)	p-value
$\hat{\alpha}(l = 1)$	0.956 (0.046)	0.955 (0.048)	0.959 (0.037)	0.022	0.957 (0.047)	0.954 (0.043)	0.220
$\hat{\alpha}(l = 2)$	0.955 (0.045)	0.955 (0.047)	0.955 (0.037)	0.950	0.956 (0.048)	0.954 (0.042)	0.100
ABI($l = 1$)	0.700 (0.169)	0.693 (0.172)	0.730 (0.152)	$< .001$	0.699 (0.171)	0.703 (0.166)	0.573
ABI($l = 2$)	0.695 (0.163)	0.690 (0.167)	0.710 (0.148)	0.008	0.691 (0.165)	0.700 (0.161)	0.148

Note: all p-values come from ANOVA test.

Table S2: Association of socio-demographic and health-related factors with standardized estimated self-similarity parameter ($\hat{\alpha}$) and activity balance index (ABI) metrics using the l parameter as 1 or 2, results from multivariate linear regressions.

	$\hat{\alpha}(l=1)$		$\hat{\alpha}(l=2)$		ABI($l=1$)		ABI($l=2$)	
	Coeff.	(95% CI)	Coeff.	(95% CI)	Coeff.	(95% CI)	Coeff.	(95% CI)
Age *per 10 years*	3.721	(2.073, 5.369)*	2.627	(0.974, 4.280)*	2.890	(1.235, 4.546)*	2.445	(0.781, 4.109)*
Age^2 *per 10 years*	-0.267	(-0.384, -0.150)*	-0.191	(-0.308, -0.073)*	-0.204	(-0.321, -0.086)*	-0.170	(-0.288, -0.052)*
Women	0.110	(0.020, 0.200)*	0.024	(-0.066, 0.113)	0.219	(0.129, 0.309)*	0.139	(0.048, 0.229)*
Currently employed	-0.178	(-0.276, -0.080)*	-0.102	(-0.201, -0.004)*	-0.101	(-0.200, -0.003)*	-0.021	(-0.120, 0.078)
High education	-0.064	(-0.140, 0.011)	-0.014	(-0.090, 0.061)	-0.076	(-0.151, 0.000)	-0.006	(-0.083, 0.070)
BMI *per 5 kg/m^2*	-0.152	(-0.194, -0.110)*	-0.171	(-0.214, -0.129)*	-0.127	(-0.169, -0.084)*	-0.133	(-0.175, -0.090)*
Number of morbidities	-0.073	(-0.123, -0.024)*	-0.078	(-0.128, -0.028)*	-0.056	(-0.106, -0.006)*	-0.050	(-0.100, 0.001)

Note: * means significant at 0.95 confidence level, estimated coefficient (Coeff.), 95% confidence interval (95% CI), high education (secondary school or above), morbidities (number of prevalent morbidities among: coronary heart disease, stroke, heart failure, cancer, arthritis, chronic obstructive pulmonary disease, depression, Parkinson's disease and dementia).

References

[1] Anderson, T. W. and Goodman, L. A. (1957) Statistical Inference about Markov Chains. Ann. Math. Stat, **28**(1), 89 – 110.
